# Bayesian blockwise inference for joint models of longitudinal and multistate data with application to longitudinal multimorbidity analysis

**DOI:** 10.1177/09622802241281959

**Published:** 2024-10-21

**Authors:** Sida Chen, Danilo Alvares, Christopher Jackson, Tom Marshall, Krish Nirantharakumar, Sylvia Richardson, Catherine L Saunders, Jessica K Barrett

**Affiliations:** 1 MRC Biostatistics Unit, 2152 University of Cambridge, Cambridge, UK; 2 Institute of Applied Health Research, 1724 University of Birmingham, Birmingham, UK; 3 Department of Public Health and Primary Care, 2152 University of Cambridge, Cambridge, Cambridgeshire, UK

**Keywords:** Bayesian joint model, electronic health records, Markov chain Monte Carlo, multimorbidity, multistate data

## Abstract

Multistate models provide a useful framework for modelling complex event history data in clinical settings and have recently been extended to the joint modelling framework to appropriately handle endogenous longitudinal covariates, such as repeatedly measured biomarkers, which are informative about health status and disease progression. However, the practical application of such joint models faces considerable computational challenges. Motivated by a longitudinal multimorbidity analysis of large-scale UK health records, we introduce novel Bayesian inference approaches for these models that are capable of handling complex multistate processes and large datasets with straightforward implementation. These approaches decompose the original estimation task into smaller inference blocks, leveraging parallel computing and facilitating flexible model specification and comparison. Using extensive simulation studies, we show that the proposed approaches achieve satisfactory estimation accuracy, with notable gains in computational efficiency compared to the standard Bayesian estimation strategy. We illustrate our approaches by analysing the coevolution of routinely measured systolic blood pressure and the progression of three important chronic conditions, using a large dataset from the Clinical Practice Research Datalink Aurum database. Our analysis reveals distinct and previously lesser-known association structures between systolic blood pressure and different disease transitions.

## 1. Introduction

Joint models (JMs) for longitudinal and time-to-event data have gained increasing interest in clinical and biomedical research.^1^ These models are instrumental in uncovering relationships between biomarkers and key health events, such as disease progression or death. Moreover, they facilitate principled, individualized predictions of these outcomes’ trajectories. Theoretical and empirical evidence shows that analysing both types of outcomes jointly leads to more accurate and efficient inference for each outcome process than analysing them separately.^2,3^

The basic formulation of JM involves a single time-to-event outcome. However, in some application contexts, more complicated event processes may arise. For instance, a subject of interest may experience a succession of intermediate events, each representing a disease/health status. Multistate models (MSMs) extend the traditional survival model to encompass more states, providing a useful probabilistic framework for modelling complex longitudinal event history data.^4^ Well-known models such as competing risks and illness-death models are special cases of MSM. MSMs have recently been extended into joint longitudinal and MSMs to allow for handling endogenous longitudinal covariates. Examples of applications of such JMs can be found by, for example, Dantan et al.^5^ for jointly studying cognitive decline, risks of dementia and death, Ferrer et al.^6^ for analysing the progression of prostate cancer, and Dessie et al.^7^ for predicting the clinical progression of HIV infection. Inference for such JMs has conventionally been based on the maximum likelihood estimation theory. More recently, Furgal^8^ explored a Bayesian estimation framework. However, we recognise that existing estimation methods face significant computational challenges with large sample sizes and when the longitudinal and/or the multistate processes become complex.^9,10^ For the frequentist approach, the main difficulty with inference arises from the numerical approximation of the integral over the random effects, which is required in computing the likelihood function. In the Bayesian approach, posterior inference is typically based on Markov chain Monte Carlo (MCMC) sampling methods, which can be computationally expensive and sometimes face difficulties with mixing and convergence. Therefore, the application of such joint longitudinal and MSMs is currently limited to relatively small sample sizes and/or simple longitudinal and multistate structures due to computational limitations.

In this article, we introduce novel Bayesian estimation approaches for joint longitudinal and MSMs for efficiently and flexibly handling complex event processes and large data sets while allowing for straightforward implementation. Instead of sampling from the joint posterior distribution of model parameters based on the entire longitudinal and multistate data, as the standard approach would do, we propose two blockwise approaches that decompose the original estimation task into smaller inference blocks. In this way, parameters associated with each block are estimated in a parallel and adaptive manner, utilizing only the longitudinal and time-to-event data pertinent to that particular block. More specifically, the first approach employs competing risk decompositions of the multistate process, estimating a joint longitudinal and competing risk model for each competing risk block. When the focus of inference and prediction lies in the multistate process, we propose a more computationally efficient blockwise inference strategy by working with each transition in the MSM individually, estimating a joint longitudinal and survival model for each transition. With blockwise approaches, model selection can be conveniently and efficiently performed in a block-specific manner using techniques such as Bayesian leave-one-out cross-validation.^11^ This facilitates the flexible specification of different models for different blocks/transitions (e.g. the association structure between the longitudinal and the multistate processes), which is a challenging task with the standard approach. Our extensive simulation studies demonstrated that the proposed approaches provide satisfactory estimation accuracy and notable efficiency gains compared to standard estimation strategy. In addition, blockwise approaches can provide more robust inference for the association and other multistate parameters in cases of model misspecification within the longitudinal process.

Multimorbidity, referred to as the co-existence of two or more long-term conditions in a single patient, is becoming increasingly prevalent and poses significant challenges to patients and public health.^12^ While previous studies have largely focused on cross-sectional patterns of multimorbidity, there is an important need to better analyse and understand the longitudinal accumulation of diseases for improved management and treatment.^13^ MSMs provide a useful framework and have been recently employed to study longitudinal patterns of multimorbidity;^14,15^ however, to our knowledge, no previous work has examined the association between longitudinal biomarkers and multimorbidity progression within a joint modelling framework. Motivated by and building on the recent study by Chen et al.,^16^ we analysed the coevolution of routinely measured systolic blood pressure (SBP) and the progression of three important chronic conditions: type-2 diabetes (T2D), mental health conditions (MH), and cardiovascular diseases (CVDs). Our analysis dataset was derived from a large electronic health records (EHRs) database in England, the Clinical Practice Research Datalink (CPRD) Aurum, which contains longitudinal routinely-collected primary care EHRs from 19 million participants across the 738 English general practices that use EMIS Web software.^17^ Notably, our proposed approaches enable us to utilize a much larger volume of patient data during inference compared to the standard inference approach. This facilitates the identification of differing association patterns between SBP and different disease transitions with enhanced statistical evidence.

The rest of the article is structured as follows. Section 2 introduces the basic Bayesian joint longitudinal and MSM and the corresponding estimation method. In Section 3, we present the proposed blockwise inference approaches, and their performance is evaluated through a simulation study in Section 4. In Section 5, we illustrate the application of the proposed approaches in a longitudinal multimorbidity analysis of large UK health records. Finally, Section 6 provides a discussion and possible directions for further work.

## 2. The Bayesian joint longitudinal and MSM

We consider a joint model consisting of longitudinal and multistate submodels, linked through shared parameters.

### 2.1. Longitudinal submodel

Let $y_{i}(t)$ be the value of the longitudinal process of subject i measured at time t , and let $y_{i}=(y_{i1},\ldots ,y_{in_{i}})$ be the observed $n_{i}$ -dimensional longitudinal response vector for the subject, where $y_{ij}=y_{i}(t_{ij})$ , $j=1,\ldots ,n_{i}$ . Typically, the longitudinal responses are modelled under a generalized linear mixed modelling (GLMM) framework. Conditional on the r -dimensional random effects vector $b_{i}$ , assumed to be independently distributed according to a normal distribution $N(0,\Sigma_{b})$ , the responses $y_{ij}$ are assumed to be independent and belong to a member of the exponential family with density

(1) $$f(y_{ij}|b_{i})=\exp\;((y_{ij}\psi_{ij}(b_{i})-c(\psi_{ij}(b_{i})))/a(\phi )-d(y_{ij},\phi ))$$

where $\psi_{ij}(b_{i})$ and ϕ denote the natural and dispersion parameters in the exponential family, respectively, and a(⋅) , c(⋅) and d(⋅) are known functions specifying the member of the exponential family. The conditional mean of $y_{ij}$ given the random effects is linked to the linear predictors via

$$E[y_{ij}|b_{i}]=c^{\prime }(\psi_{ij})=g^{-1}(X_{i}^{T}(t_{ij})\beta +Z_{i}^{T}(t_{ij})b_{i})$$

where g(⋅) denotes a known monotonic link function, β is a $q_{1}$ -dimensional fixed effects vector, and $X_{i}(t)$ and $Z_{i}(t)$ denote the possibly time-dependent design vectors for the fixed and random effects, respectively. In this article, motivated by our case study, we focus on continuous longitudinal responses, in which case the GLMM reduces to a standard Gaussian linear mixed model (LMM)

(2) $$y_{ij}|b_{i}∼N(\mu_{i}(t_{ij}|b_{i}),\sigma_{e}^{2})$$

where $\mu_{i}(t_{ij}|b_{i})=X_{i}^{T}(t_{ij})\beta +Z_{i}^{T}(t_{ij})b_{i}$ and $\sigma_{e}^{2}$ is the error variance. The collection of parameters associated with the longitudinal submodel is denoted by $\theta^{L}=(\beta ,\sigma_{e}^{2},\Sigma_{b})$ .

### 2.2. Multistate submodel

Let $\left\{E_{i}(t),t\geq 0\right\}$ be a continuous-time multistate process for subject i with a common state space S={0,1,…,N} , where $E_{i}(t)$ denotes the state that subject i occupies at time t . Let S={(j,k):j,k∈S;j≠k;j→kisa permitted direct transition} . The evolution of the process $E_{i}(t)$ can be fully characterized by the transition intensities

(3) $$h_{jk}^{\left(i\right)}(t|H_{t^{-}})=\lim_{\Delta t\to 0}\frac{P(E_{i}(t+\Delta t)=k|E_{i}(t)=j;H_{t^{-}})}{\Delta t},\;(j,k)\in S$$

which represents the instantaneous risk of transiting from state j to state k at time t given the history up to time t , $H_{t^{-}}$ . In this article, motivated by our real data application, we focus on unidirectional MSMs, that is, transitions between states can only occur in one direction. The regression models for the transition intensities are commonly specified in the form

(4) $$h_{jk}^{\left(i\right)}(t|H_{t^{-}})=h_{0,jk}(t|H_{t^{-}};\phi_{jk})\exp\;(w_{i}^{T}\gamma_{jk}+f(\beta ,b_{i},t,\alpha_{jk}))$$

with $h_{0,jk}(\cdot )$ representing a baseline intensity function parameterized by $\phi_{jk}$ and $w_{i}$ be the $q_{2}$ -dimensional vector of exogenous risk factors associated with the coefficient vector $\gamma_{jk}$ . The collection of parameters associated with the transition j→k in the multistate submodel is denoted by $\theta_{jk}^{E}=(\phi_{jk},\gamma_{jk},\alpha_{jk})$ . Depending on the application context, it is common to impose either a Markov (i.e. $h_{0,jk}(t|H_{t^{-}})=h_{0,jk}(t)$ ) or a semi-Markov assumption (i.e. $h_{0,jk}(t|H_{t^{-}})=h_{0,jk}(B(t))$ , where B(t) is the time since entry into the current state j ) on the transition intensities to simplify the dependence structure on the history of the process.^18^ The function f , parametrized by $\alpha_{jk}$ , describes the association between the longitudinal marker’s dynamics and the multistate process. Some common choices of f are $f=\alpha_{jk}\mu_{i}(t)$ (current value association), $f=\alpha_{jk}\mu_{i}^{\prime }(t)$ (current slope association) or $f=\alpha_{jk}^{T}b_{i}$ (shared random effects). In particular, $\alpha_{jk}$ quantifies the strength of the association between the two processes.

To formally define the model likelihood, we assume that each subject is followed up continuously for some period of time, subject to a right censoring time $C_{i}$ , and let $E_{i}=\{E_{i}(t),0\leq t\leq C_{i}\}$ be the observed multistate process for subject i . Let $\left(T_{1}^{\left(i\right)},\ldots ,T_{N_{i}}^{\left(i\right)}\right)$ be the sequence of observed transition times such that $T_{l}^{\left(i\right)}<T_{l+1}^{\left(i\right)}$ ; $N_{i}$ is the total number of observed transitions ( $N_{i}=0$ if the subject is censored without having a transition), and let $T_{0}^{\left(i\right)}=0$ and $T_{N_{i}+1}^{\left(i\right)}=C_{i}$ . We additionally define the transition indicator variables $\delta_{l,jk}^{\left(i\right)}$ such that $\delta_{l,jk}^{\left(i\right)}=1$ if the transition j→k occurs at event time $T_{l}^{\left(i\right)}$ and $\delta_{l,jk}^{\left(i\right)}=0$ otherwise. With the notation and assumptions above, the likelihood contribution of the multistate process for subject i can be expressed as follows:

(5) $$\begin{array}{rl} f(E_{i}|\cdot ) & =\prod_{1\leq l\leq N_{i}+1}\left(\prod_{j,k:(j,k)\in S}h_{jk}^{\left(i\right)}(T_{l}^{\left(i\right)}{)}^{\delta_{l,jk}^{\left(i\right)}}\right)\\ & \;\times \exp\;\left(-\int_{T_{l-1}^{\left(i\right)}}^{T_{l}^{\left(i\right)}}\sum_{j\in S}h_{E_{i}(T_{l-1}^{\left(i\right)}),j}^{\left(i\right)}(u)du\right) \end{array}$$

where the intensity functions $h_{jk}^{\left(i\right)}(\cdot )$ are given by (4) (for brevity, here and in what follows we omit the conditioned history and parameter set) and we adopt the convention that $0^{0}$ is taken to be one. A rigorous derivation of the likelihood can be made based on the counting process theory, see Cook and Lawless^4^ for details. Note that in general integrals in (5) cannot be computed analytically and numerical approximation is required. In our implementation, we used the Gaussian-Legendre quadrature method (with 15 quadrature points).

### 2.3. Bayesian inference for the joint longitudinal and MSM

Assuming that the longitudinal and multistate processes are conditionally independent given the random effects, as adopted in earlier literature,^6^ the posterior distribution of the model parameters and random effects can be factorised as follows:

(6) $$\begin{array}{rl} \pi (\Theta ,b|D_{\text{full}}) & \propto L(\Theta ,b|D_{\text{full}})f(b|\Theta )\pi (\Theta )\\ & =\left(\prod_{i=1}^{n}\prod_{j=1}^{n_{i}}f(y_{ij}|b_{i},\Theta )f(E_{i}|b_{i},\Theta )\right)\left(\prod_{i=1}^{n}f(b_{i}|\Theta )\right)\pi (\Theta ) \end{array}$$

where $\Theta =(\theta^{L},\theta^{E})$ is the full model parameter vector with $\theta^{E}=\{\theta_{jk}^{E}\}$ , $b=(b_{1},\ldots ,b_{n})$ and $D_{\text{full}}$ represents the full dataset. The conditional densities in (6) are given in (1) and (5). To complete the Bayesian formulation, the joint prior distribution for all the model parameters, π(Θ) , needs to be specified. In practice, independent and weakly informative prior distributions are commonly used for parameters in JMs.^19,20^ The posterior defined in (6) is not analytically tractable. MCMC methods can be employed for inference purposes.

## 3. Blockwise parallel inference

**Figure 1.**
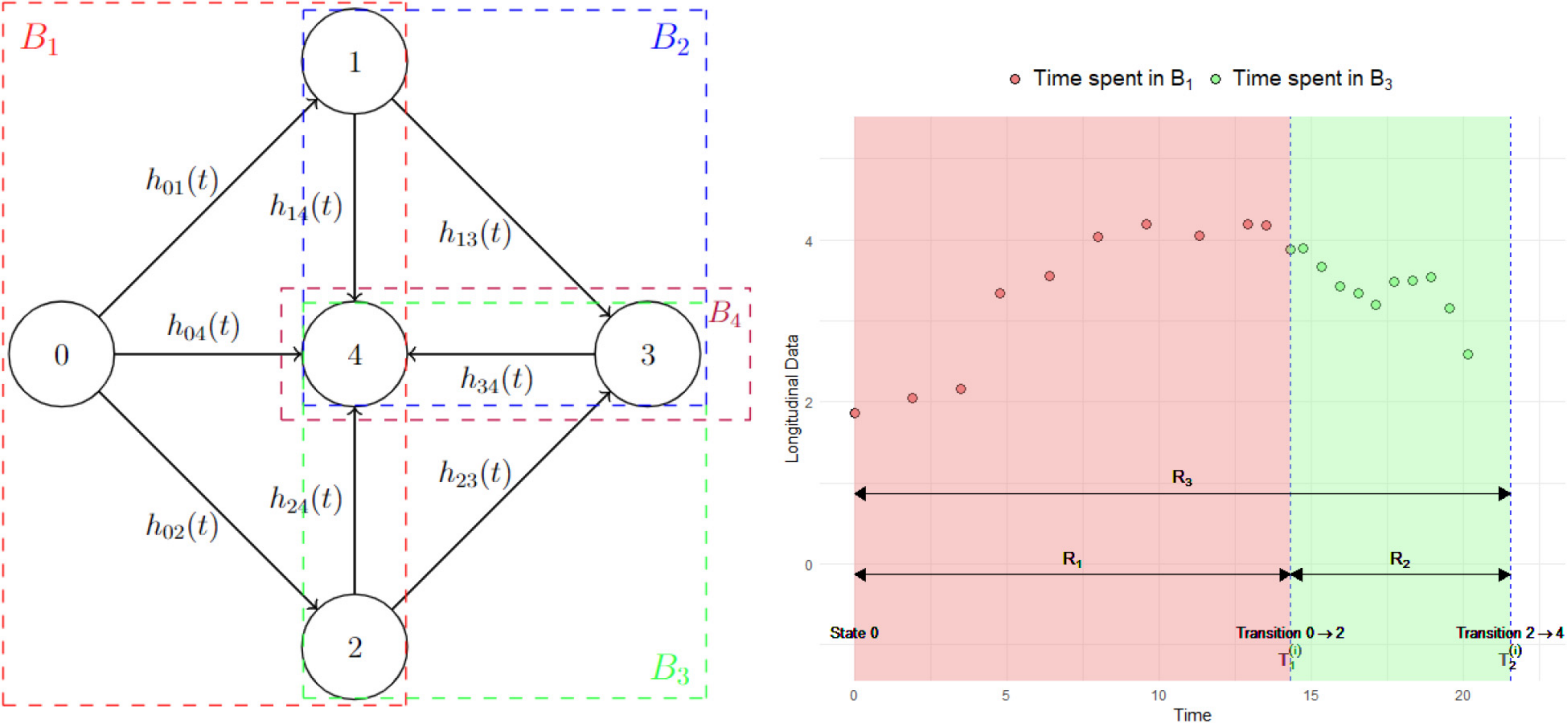
Illustration of the blockwise inference approach. Left panel: multistate progressive scheme with its transition blocks. A total of eight different transitions are allowed: 0→1 , 0→2 , 0→4 , 1→3 , 1→4 , 2→3 , 2→4 and 3→4 with corresponding transition intensities $h_{01}(t)$ , $h_{02}(t)$ , $h_{04}(t)$ , $h_{13}(t)$ , $h_{14}(t)$ , $h_{23}(t)$ , $h_{24}(t)$ and $h_{34}(t)$ . For the JM-CR approach there are four blocks: $B_{1}$ , $B_{2}$ , $B_{3}$ and $B_{4}$ , as indicated by coloured dashed boxes. The JM-ST approach has eight blocks, corresponding to each permitted transition. Right panel: hypothetical longitudinal profile of a marker over time for an imaginary subject experiencing transition 0→2 at $T_{1}^{\left(i\right)}$ and transition 2→4 at $T_{2}^{\left(i\right)}$ . The data collected during the time spent in $B_{1}$ (indicated by $R_{1}$ ) and $B_{3}$ (indicated by $R_{2}$ ) are indicated with red and green shades, respectively. The entire follow-up period for this subject is indicated by $R_{3}$ . JM-CR: joint longitudinal and competing risk; JM-ST: joint longitudinal and single transition.

In this section, we describe the proposed intuitive blockwise inference approaches to alleviate the computational challenges of fitting the joint longitudinal and MSM (JM-MSM) described in Section 2. To help illustrate the idea, we employ an example throughout as shown in Figure 1. A high-level comparison of the standard and proposed inference approaches is provided in Table 1, whereas a more detailed comparison of the asymptotic and finite sample behaviours of these approaches is provided in Supplemental Section A.

**Table 1.** Comparing the standard (JM-MSM) and blockwise approaches (JM-CR and JM-ST) in terms of data used in the posterior computation, with notations defined as in Sections 2 and 3. $B_{v}$ represents a generic competing risk block, and $B_{j_{v},k}$ denotes a generic transition within the block. $I_{B_{v}}$ and $T_{B_{v}}^{\left(i\right)}$ , respectively, indicate the index sets of subjects entering $B_{v}$ and the longitudinal data points used for inference in $B_{v}$ for subject $i\in I_{B_{v}}$ . $D_{B_{v}}$ and $D_{B_{j_{v},k}}$ are subsets of the full dataset, $D_{\text{full}}$ .

	Individual-level data			
Approach	Time-to-event data	Longitudinal data	Subjects involved	Dataset
JM-MSM	Multistate data	All data	All subjects	$D_{\text{full}}$
	$E_{i}$	$y_{i}$
JM-CR	Competing risk data	Block-specific subset	Block-specific subset	$D_{B_{v}}$
	$E_{i}^{B_{v}}$	$\{y_{ij}{\}}_{j\in T_{B_{v}}^{\left(i\right)}}$	$i\in I_{B_{v}}$
JM-ST	Survival data	Block-specific subset	Block-specific subset	$D_{B_{j_{v},k}}$
	$E_{i}^{B_{j_{v},k}}$	$\{y_{ij}{\}}_{j\in T_{B_{v}}^{\left(i\right)}}$	$i\in I_{B_{v}}$

JM-MSM: joint longitudinal and multistate model; JM-CR: joint longitudinal and competing risk; JM-ST: joint longitudinal and single transition.

### 3.1. The joint longitudinal and competing risks (JM-CRs) approach

Our first proposal involves breaking down the multistate process into separate connected blocks of competing risk processes. This decomposition is unique and we utilize this to fit a JM-CR model to each block independently, using only the longitudinal and time-to-event data that are associated with the block. In the example shown in Figure 1, the multistate process can be decomposed into four competing risk blocks, $B_{1}$ , $B_{2}$ , $B_{3}$ and $B_{4}$ . Consider a generic competing risk block $B_{v}$ . Let $I_{B_{v}}$ denote the index set of subjects who entered the initial state in $B_{v}$ and were at risk for transitions in $B_{v}$ . Let $T_{B_{v}}^{\left(i\right)}$ denote the index set for time point j such that $\{y_{ij}{\}}_{j\in T_{B_{v}}^{\left(i\right)}}$ represents the longitudinal data linked to block $B_{v}$ for subject i (see Section 3.3 for more details on the construction of this index set), $E_{i}^{B_{v}}$ be the observed competing risk process associated with $B_{v}$ , and $S_{B_{v}}$ be the set of the indices of the terminal state in $B_{v}$ . Then we define the likelihood function for the JM-CR approach for $B_{v}$ as

(7) $$L(\Theta_{B_{v}},b_{B_{v}}|D_{B_{v}})=\prod_{i\in I_{B_{v}}}\prod_{j\in T_{B_{v}}^{\left(i\right)}}f(y_{ij}|b_{i},\Theta_{B_{v}})f(E_{i}^{B_{v}}|b_{i},\Theta_{B_{v}})$$

where $\Theta_{B_{v}}$ and $b_{B_{v}}$ are vectors of the associated model parameters and random effects, $D_{B_{v}}$ denotes the total conditioning data for inference in block $B_{v}$ and

(8) $$f(E_{i}^{B_{v}}|\cdot )=\prod_{k\in S_{B_{v}}}h_{j_{v}k}^{\left(i\right)}(T_{l_{i,B_{v}}}^{\left(i\right)}{)}^{\delta_{j_{v}k}^{\left(i\right)}}\exp\;\left(-\int_{T_{l_{i,B_{v}}-1}^{\left(i\right)}}^{T_{l_{i,B_{v}}}^{\left(i\right)}}\sum_{k\in S_{B_{v}}}h_{j_{v},k}^{\left(i\right)}(u)du\right)$$

where $j_{v}$ denotes the initial state index in $B_{v}$ , $\delta_{jk}^{\left(i\right)}$ is the transition indicator variable defined analogously as before indicating if the individual makes a transition from j→k and $l_{i,B_{v}}\in \{1,\ldots ,N_{i}+1\}$ such that $T_{l_{i,B_{v}}}^{\left(i\right)}$ denotes the observed transition time within $B_{v}$ or the right censoring time. Posterior inference under JM-CR is performed by sampling from $f(\Theta_{B_{v}},b_{B_{v}}|D_{B_{v}})$ independently for each block instead of sampling from (Θ,b) jointly at once (see also Table 1).

### 3.2. The joint longitudinal and single transition (JM-ST) approach

The JM-ST approach treats each allowed transition j→k as a block, denoted by $B_{j,k}$ , and estimates a joint longitudinal and survival model independently for each such block. Consider a generic competing risk block $B_{v}$ and let $E_{i}^{B_{j_{v},k}}$ denote the observed survival process for subject i considering the transition $j_{v}\to k$ , $k\in S_{B_{v}}$ (i.e. subjects who transition to a competing state or are censored are both treated as censored). We define the working likelihood function of the JM-ST approach for $B_{j_{v},k}$ as

(9) $$L(\Theta_{B_{j_{v},k}},b_{B_{j_{v},k}}|D_{B_{j_{v},k}})=\prod_{i\in I_{B_{v}}}\prod_{j\in T_{B_{v}}^{\left(i\right)}}f(y_{ij}|b_{i},\Theta_{B_{j_{v},k}})f(E_{i}^{B_{j_{v},k}}|b_{i},\Theta_{B_{j_{v},k}})$$

where $\Theta_{B_{j_{v},k}}$ and $b_{B_{j_{v},k}}$ are vectors of the transition-specific model parameters and random effects, respectively. $D_{B_{j_{v},k}}$ denotes the data for inference in $B_{j_{v},k}$ . The index sets $I_{B_{v}}$ and $T_{B_{v}}^{\left(i\right)}$ are defined as in the JM-CR approach, and $f(E_{i}^{B_{j_{v},k}}|\cdot )$ is given by

(10) $$f(E_{i}^{B_{j_{v},k}}|\cdot )=h_{j_{v}k}^{\left(i\right)}(T_{l_{i,B_{v}}}^{\left(i\right)}{)}^{\delta_{j_{v}k}^{\left(i\right)}}\exp\;\left(-\int_{T_{l_{i,B_{v}}-1}^{\left(i\right)}}^{T_{l_{i,B_{v}}}^{\left(i\right)}}h_{j_{v},k}^{\left(i\right)}(u)du\right)$$

which corresponds to a standard survival likelihood, with $T_{l_{i,B_{v}}}^{\left(i\right)}$ defined as in (8). Therefore, when using the JM-ST approach to estimate each transition, $B_{j_{v},k}$ , the same subset of subjects ( $I_{B_{v}}$ ) is used as in the JM-CR approach for estimating block $B_{v}$ , and the same set of longitudinal data is used if the same index set $T_{B_{v}}^{\left(i\right)}$ is adopted (see Table 1). Posterior inference under JM-ST is performed by sampling from $f(\Theta_{B_{j_{v},k}},b_{B_{j_{v},k}}|D_{B_{j_{v},k}})$ independently for each transition. Note that the JM-ST approach is applicable when the focus of inference or prediction is on the multistate process. Unlike the JM-MSM or JM-CR approaches, it does not allow estimating individual-specific trajectories for the longitudinal marker as subjects are reused to estimate transitions within a competing risk block, resulting in multiple sets of longitudinal parameter and random effect estimates per subject.

### 3.3. Incorporating longitudinal data in model blocks

When applying the JM-CR or JM-ST approach to perform inference in a specific block, we need to specify the index set $T_{B_{v}}^{\left(i\right)}$ , which determines the amount of longitudinal data used for estimating the longitudinal trajectories. In principle, assuming that the longitudinal submodel is correctly specified, we could achieve increasingly accurate inference as the cardinality of $T_{B_{v}}^{\left(i\right)}$ increases (see also the discussion in Supplemental Section A). However, in practice, the correct specification assumption may not hold, so there is a bias-variance trade-off between the amount of data used and potential misspecification bias. In this article, we consider two natural proposals. The first is to always use all available historical data collected since the start of the follow-up. In the example shown in Figure 1, this corresponds to using both the red and green shaded data (data collected during $R_{3}$ ) when using JM-CR for $B_{3}$ or JM-ST for transitions 2→3 and 2→4 . This strategy could be particularly useful if most subjects have little or no longitudinal data within the block, as it allows borrowing information from historical data. The second strategy is to use only the concurrent data collected during the time subjects spent in the given block (if no data are available within the block for a subject, historical data will be used). Referring again to the example in Figure 1, this means that only the green shaded data (data collected during $R_{2}$ ) would be used for inference in $B_{3}$ when applying JM-CR, or for transitions 2→3 and 2→4 when using JM-ST. In our experience, the latter often works well with a moderate amount of longitudinal data as it allows a more adaptive modelling of the marker’s trajectory. A more formal comparison between these two options can be conducted within the Bayesian inferential framework using the criterion described in Section 3.4.

### 3.4. Model comparison

To compare candidate models for the same data, we consider a Bayesian leave-one-out cross-validation (LOO-CV) score as suggested by Vehtari et al.^11^ for approximating the predictive accuracy of a fitted Bayesian model. The LOO-CV score is defined as follows:

(11) $$\text{LOO-CV}=\sum_{i=1}^{n}\log\;f(D_{i}|D_{-i}^{\left(n\right)})$$

where $D_{i}$ denotes the i th observation, $D^{\left(n\right)}=(D_{1},\ldots ,D_{n})$ and $D_{-i}^{\left(n\right)}$ represents the dataset excluding the i th observation. Note that $f(D_{i}|D_{-i}^{\left(n\right)})=\int f(D_{i}|\theta ,D_{-i}^{\left(n\right)})f(\theta |D_{-i}^{\left(n\right)})d\theta$ , where θ is a vector of model parameters, measures how well the model would predict the i th observation based on the model estimated using the data without including that observation. The LOO-CV can be effectively estimated based on existing simulated MCMC samples using a Pareto smoothed importance sampling approach proposed by Vehtari et al.,^11^ which is implemented in the R package loo,^21^ avoiding the need to refit the model n times. In this joint modelling context, it is natural for the i th observation $D_{i}$ to include both longitudinal and time-to-event data; that is, $D_{i}=(y_{i},E_{i}^{B_{v}})$ for JM-CR, or $D_{i}=(y_{i},E_{i}^{B_{j_{v},k}})$ for JM-ST. Model selection can be performed in a blockwise manner when implementing the JM-CR or JM-ST approach. For instance, with JM-ST, for each transition, different candidate models are fitted and a model can be selected based on the LOO-CV computed for the data associated with that transition.

## 4. Simulation study

To thoroughly evaluate the accuracy and computational efficiency of our proposed blockwise approaches for inference in Bayesian joint longitudinal and MSMs, we conducted simulation studies under both realistic and hypothetical scenarios. For each of the JM-CR and JM-ST approaches, we explored two different configurations as outlined in Section 3.3: one using solely concurrent longitudinal data (JM-CR-C and JM-ST-C) and another utilizing all available historical longitudinal data (JM-CR-H and JM-ST-H) for inference in a given block. The benchmark comparator is the standard JM-MSM approach described in Section 2. For all approaches, the same set of weakly informative priors was used, and posterior sampling was conducted using Rstan^22^ with the no-U-turn sampler (NUTS),^23^ a state-of-the-art MCMC algorithm.

### 4.1. Simulation models

Here we briefly describe the settings for the two main simulation models, which are motivated by two different real data applications.

#### 4.1.1. Model 1

Model 1 is derived from our case study in Section 5 for analysing longitudinal multimorbidity progression. The multistate process is shown in Figure 1. We assume clock-reset semi-Markov transition dynamics with the transition intensities specified as follows:

(12) $$h_{jk}^{\left(i\right)}(t|H_{t^{-}})=h_{0,jk}(B(t))\exp\;(w_{i}\gamma_{jk}+\alpha_{jk}\mu_{i}(t))$$

where (j,k)∈{(0,1),(0,2),(0,4),(1,3),(1,4),(2,3),(2,4),(3,4)} , $h_{0,jk}(u)=\delta_{jk}u^{\delta_{jk}-1}\lambda_{jk}$ is the Weibull hazard function specified by the shape parameter $\delta_{jk}$ and scale parameter $\lambda_{jk}$ , and $w_{i}$ is a baseline covariate for subject i and $\mu_{i}(t)$ is the unobserved longitudinal trajectory for subject i at time t . For the longitudinal process, we consider a basic LMM with a random intercept and slope:

(13) $$y_{i}(t)=\mu_{i}(t)+\epsilon_{i}(t),\;\epsilon_{i}(t)∼N(0,\sigma_{e}^{2})$$

with $\mu_{i}(t)=\beta_{1}+b_{i1}+(\beta_{2}+b_{i2})t$ and $\left(b_{i1},b_{i2})∼N(0,\Sigma_{b}\right)$ .

#### 4.1.2. Model 2

Model 2 is derived from Ferrer et al.,^6^ where the authors use the model to analyse the progression of prostate cancer. The associated multistate transition diagram extends that in Figure 1 by adding two additional transitions 0→3 and 1→2 (see Figure 3 by Ferrer et al.^6^). For this model, we can identify four blocks for the JM-CR approach and 10 blocks for the JM-ST approach. The regression models for the transition intensities are specified with the Markov assumption and a clock forward time scale as

(14) $$h_{jk}^{\left(i\right)}(t|H_{t^{-}})=h_{0,jk}(t)\exp\;(w_{i}\gamma_{jk}+\alpha_{jk}\mu_{i}(t))$$

where (j,k)∈{(0,1),(0,2),(0,3),(0,4),(1,2),(1,3),(1,4),(2,3),(2,4),(3,4)} . The baseline intensity functions are modelled using cubic B-splines as

(15) $$h_{0,jk}(t)=\exp\;\left(\sum_{l=1}^{5}\eta_{jk,l}B_{l}(t)\right),\;(j,k)\in \{(0,1),(0,3),(0,4)\}$$

where the $B_{l}(t)$ are pre-specified cubic B-spline basis functions and $\eta_{jk,l}$ are the associated spline coefficients. Baseline intensities for other transitions are proportional to these transitions via re-scaling. The longitudinal data are generated from a LMM exhibiting a non-linear trend, with

(16) $$\begin{array}{rl} \mu_{i}(t) & =\beta_{1}+\beta_{1,w}w_{i}+b_{i1}+(\beta_{2}+\beta_{2,w}w_{i}+b_{i2})f_{1}(t)\\ & \;+(\beta_{3}+\beta_{3,w}w_{i}+b_{i3})f_{2}(t) \end{array}$$

where $f_{1}(t)=(1+t{)}^{-1.2}-1$ , $f_{2}(t)=t$ , $\left(b_{i1},b_{i2},b_{i3})∼N(0,\Sigma_{b}\right)$ and the β s are fixed effect parameters.

**Figure 2.**
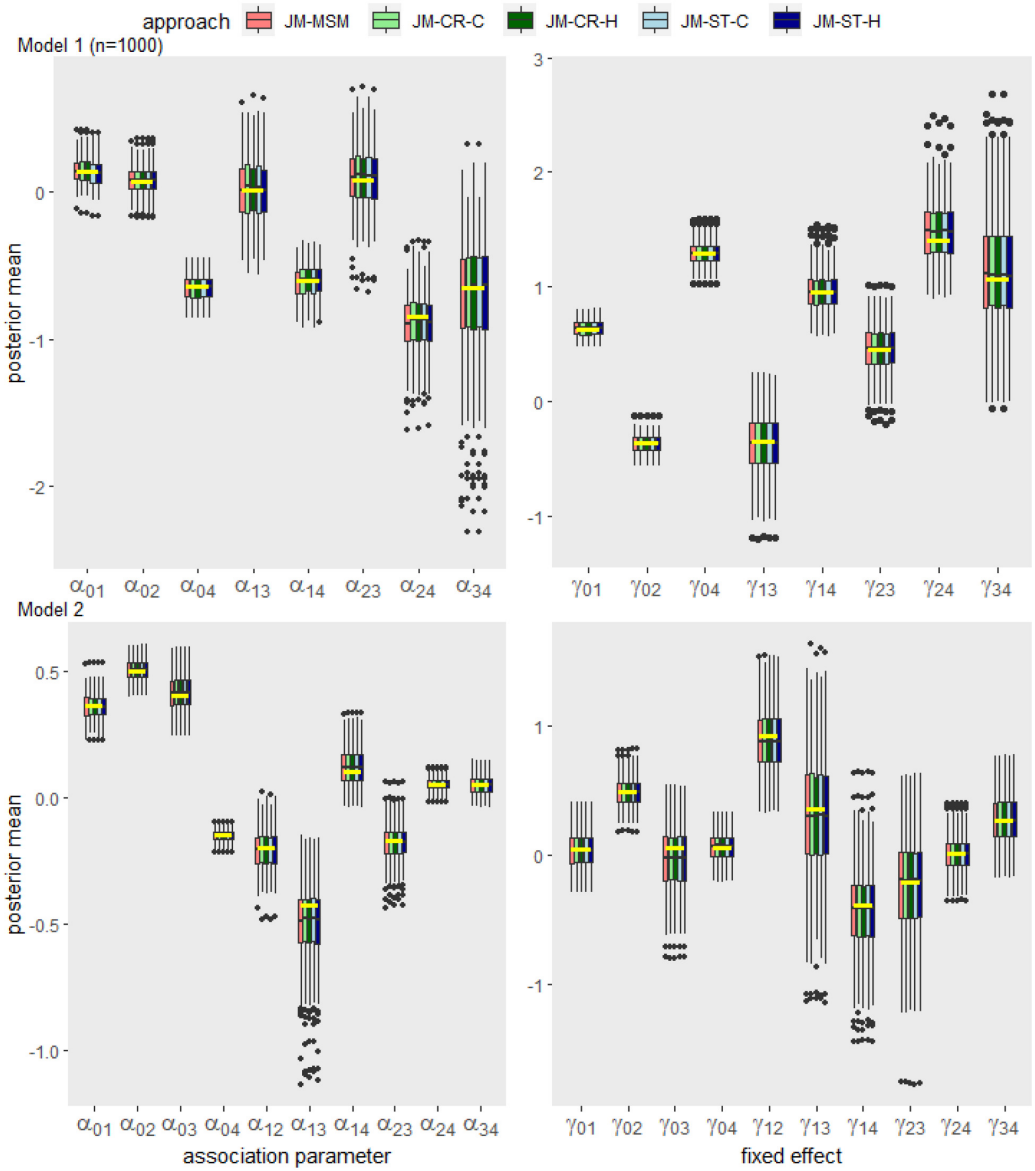
Box plot summary of the posterior means of the association and fixed effect parameters obtained by each approach across 200 data replications for Model 1 ( n=1000 ; upper panel) and Model 2 ( n=900 ; lower panel). The true values for each parameter are indicated by yellow horizontal bars.

**Figure 3.**
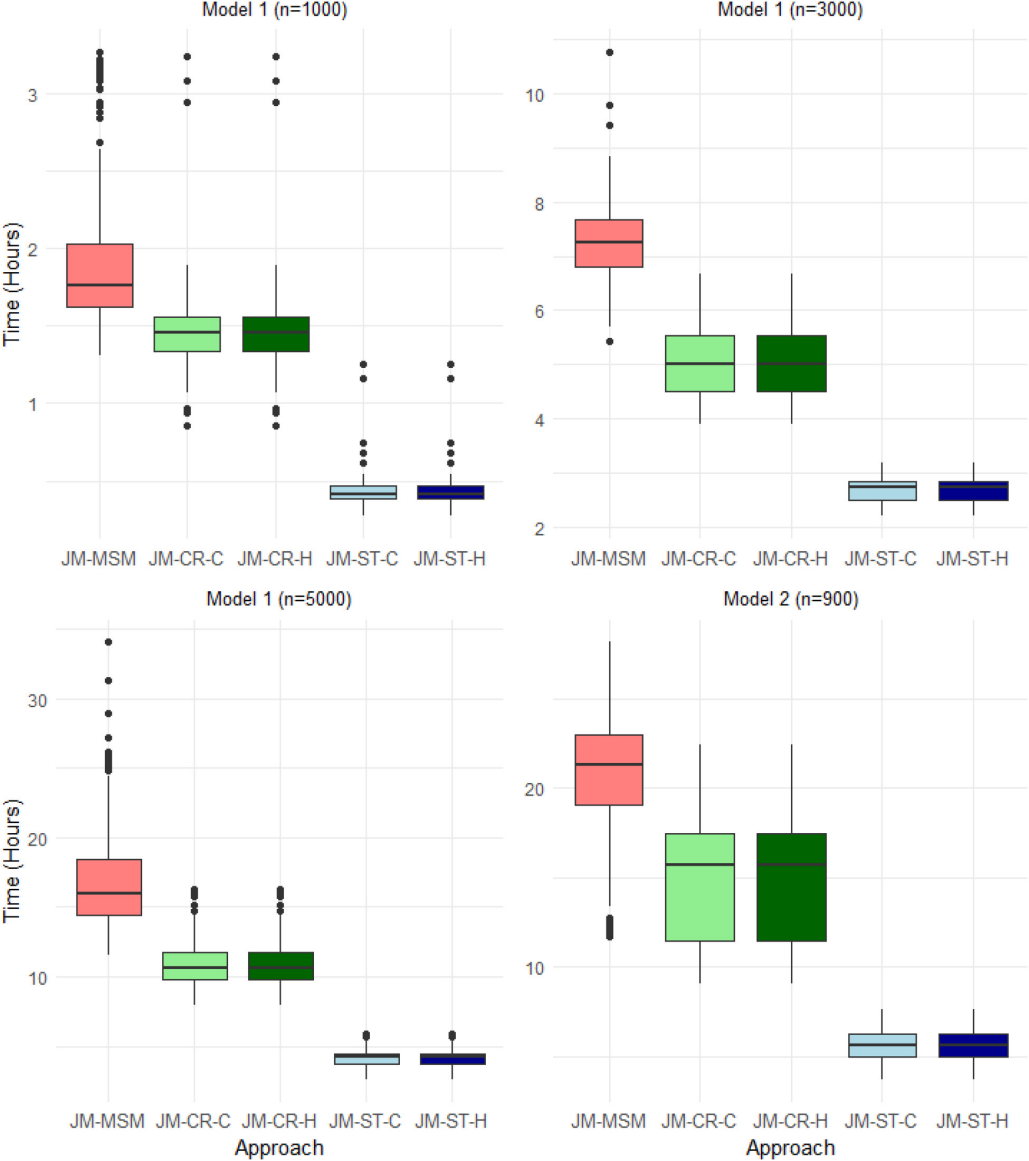
Box plot summary of the total computation times required to obtain 1000 convergent Markov chain Monte Carlo (MCMC) samples, including the time spent in the burn-in period, for each approach across 200 replications of the data for Models 1 (upper left, upper right and lower left panels) and 2 (lower right panel). For all cases and approaches, parameters were initialized at zero on the transformed unconstrained space in Rstan.

### 4.2. Data generation and prior settings

We use the following parameter settings to simulate data from the simulation models. For Model 1, $w_{i}$ is considered as an artificial ‘age’ covariate, generated from a mixture of uniform distributions defined over the intervals (18,65) , (65,80) and (80,90) with weights 0.55 , 0.3 and 0.15 , respectively. The variable is then normalized to have a mean of 0 and a standard deviation of 1. For each subject, we generate a random right censoring time from a uniform distribution, $C_{i}∼U(13,24)$ . To generate the visiting time points $t_{ij}$ , we set an equidistant sampling interval of $\Delta_{1}=2.6$ before the first transition, and decrease it to $\Delta_{2}=2$ and $\Delta_{3}=1.2$ after the first and second transitions, respectively. To set the transition-specific parameters in the multistate submodel (i.e. $\left\{\gamma_{jk}\right\}$ , $\left\{\alpha_{jk}\right\}$ , $\left\{\delta_{jk}\right\}$ and $\left\{\lambda_{jk}\right\}$ ), we fit a separate JM for each transition using the JM-ST-C approach based on a random subset of subjects’ data from the CPRD dataset. Parameters in the longitudinal submodel are obtained by fitting a LMM (as specified in (13)) on the standardised log-transformed SBP based on subjects in the CPRD dataset who has had a diagnosis of T2D. For Model 2, we set $C_{i}∼U(4,24)$ , and the visiting time points $t_{ij}$ are generated with an equidistant gap of Δ=1 . All other model parameters are based on the setting by Ferrer et al.,^6^ and we refer to their paper for details.

Given the parameter values, a joint longitudinal and multistate process can be simulated according to the generative process of the model. Firstly, subject-specific random effects $b_{i}$ are simulated independently from their prior distribution $N(0,\Sigma_{b})$ , and for each subject i=1,…,n , we generate a right censoring time point $C_{i}$ (either randomly or deterministically). Conditional on the $b_{i}$ , we simulate the multistate process $E_{i}(t)$ for $0\leq t\leq C_{i}$ using the scheme as described by, for example, Crowther et al.^24^ Then, the longitudinal trajectory is simulated by first generating the measurement time points $t_{ij}$ during the follow-up period $0\leq t\leq C_{i}$ up to reaching the terminal absorbing state. Subsequently, the data $y_{i}(t_{ij})$ are generated according to the specified longitudinal submodel.

In our implementation, we used the following set of weakly informative and independent prior distributions for all approaches and simulation scenarios: $\alpha_{jk},\gamma_{jk},\beta_{i},\beta_{i,w}∼N(0,100^{2})$ ; $\eta_{jk,i}∼N(0,10^{2})$ ; $\lambda_{jk},\delta_{jk}∼\text{half-Cauchy}(0,1)$ ; and $\sigma_{e}^{2}∼\text{Inv-Gamma}(0.01,0.01)$ ; where half-Cauchy(μ,σ) denotes a half-Cauchy distribution with location parameter μ and scale parameter σ and Inv-Gamma(α,β) denotes an inverse Gamma distribution with shape parameter α and scale parameter β . For the covariance matrix of the random effects, we followed Stan’s recommendation to decompose it into a vector of standard deviations and a correlation matrix. Each standard deviation was assigned a half-Cauchy(0,2.5) prior, and the correlation matrix was assigned a LKJ(2) prior.^25^ In our experiments, the results were insensitive to the choice of these priors.

### 4.3. Results

For Model 1, we examined three different sample sizes: n=1000 , n=3000 and n=5000 ; and for Model 2, we fixed n=900 , considering the constraints of available computational resources. In each case, we generated N=200 independent replications of the dataset. For the JM-CR and JM-ST approaches, parameters within each block were sampled from the respective posteriors in parallel, and the computation time was determined by taking the maximum computing time across all blocks. Posterior summaries were derived based on 1000 samples generated by NUTS, using default control parameters. A suitable burn-in period, $T_{b}$ , was determined based on preliminary runs and convergence was assessed based on the trace plots and the scale reduction statistic $\hat{R}$ . For Model 1, we set $T_{b}=300$ for n=1000 and $T_{b}=500$ for larger sample sizes and for Model 2 we set $T_{b}=400$ . All computations were performed on the Cambridge Service for Data Driven Discovery (CSD3) High-Performance Computing (HPC) system using the Ice Lake CPUs.

Figure 2 summarizes the estimated posterior means of the association and fixed effect parameters obtained by each approach across 200 data replications for Model 1 ( n=1000 ) and Model 2. For both models, the distributions of the point estimates obtained from the JM-CR and JM-ST approaches align with those from the JM-MSM approach, which is based on the ground truth model that generated the simulation data. Supplemental Figures 1 to 4 show additional estimation results for Model 1, including cases with larger sample sizes. As the sample size n increases, the estimation accuracy of all five approaches improves, and variability of the estimates from all approaches decreases at a similar rate. Supplemental Tables 1 to 5 show the coverage probability (the proportion of times that the credible interval contains the true value of the parameter in repeated simulations) of the estimated 95% credible intervals for the association and other MSM parameters obtained by each approach across the 200 data replications for Models 1 and 2. In all scenarios, all approaches yielded similar coverage probabilities with slight variations around the theoretical value of 0.95 .

Figure 3 shows the comparison of the total computation times, including the burn-in period, required by the five approaches to obtain 1000 convergent MCMC samples for Models 1 and 2. As expected, the JM-ST approach is the most efficient in all scenarios, while the JM-MSM approach requires the longest computation time. For the blockwise approaches, the two sub-versions (JM-CR-C/JM-ST-C and JM-CR-H/JM-ST-H) have almost the same computational costs. Additional experiments suggest that the initialization strategy of the sampler and the sample size (not shown here) appear to have a more substantial impact on the convergence of the JM-MSM approach compared to the blockwise approaches. With random or overdispersed initialization, or with increasing sample sizes, JM-MSM typically requires a longer burn-in period to reach convergence, whereas JM-ST is the least affected.

We performed additional simulations building on Model 1 to examine the performance of the blockwise approaches in the presence of misspecified longitudinal trajectory or multistate transition dynamics, and the details are provided in Supplemental Section B. In brief, the blockwise approaches can offer greater robustness in the former scenario (see Supplemental Figure 5) and do not exhibit greater estimation bias than the JM-MSM approach in the latter scenario (see Supplemental Figure 6).

## 5. Application to the CPRD data

Here we illustrate the use of the proposed approaches by analysing the association between routinely measured SBP and the progression of multimorbidity defined as the combinations of T2D, MH and CVD. We used the CPRD Aurum cohort considered by Chen et al.,^16^ which comprises electronic primary care records from 13.48 million participants in England. The data span from 2005 to 2020, with a median follow-up of 4.71 years (IQR: 1.78–11.28). In this analysis, we focused on subjects with an initial T2D diagnosis (among the three conditions) and had at least one SBP measurement recorded during their follow-up period (any SBP measurements taken within 3 months before death were removed to reduce the potential confounding effects of the near-death period on the SBP). We modelled the standardized log-transformed SBP using a LMM, incorporating a random intercept and slope as in equation (13). The disease progression was modelled using the multistate process depicted in Figure 1, where state 0 represents the T2D diagnosis at baseline, states 1, 2 and 3 represent disease combinations T2D+CVD, T2D+MH and T2D+CVD+MH, respectively. The terminal absorbing state, state 4, denotes the death state. The transition intensities, assuming a clock-reset timescale and a current value association structure between SBP and disease transition rate, are specified as follows:

(17) $$h_{jk}^{\left(i\right)}(t|H_{t^{-}})=h_{0,jk}(B(t))\exp\;(w_{i}^{T}\gamma_{jk}+\alpha_{jk}\mu_{i}(t))$$

where for modelling flexibility, we follow Ferrer et al.^6^ to specify baseline intensities using cubic B-splines as $h_{0,jk}(t)=\exp\;(\sum_{l=1}^{5}\eta_{jk,l}B_{jk,l}(t))$ , with $B_{jk,l}(t)$ being the l -th basis function defined with boundary knots at 0 and 16 and one internal knot placed at the median of the associated observed transition times. For the baseline risk factors $w_{i}$ , motivated by the analysis by Chen et al.,^16^ we considered four important sociodemographic characteristics associated with multimorbidity progression: ethnicity groups (White, South Asian, Black, Mixed, and Others), social and material deprivation (approximated using the English Index of Multiple Deprivation (IMD)), age at entry to the current state (in decades), and sex (1: male; 0: female). Results by Chen et al.^16^ suggest a significant imbalance among the non-White groups, who generally have a hazard ratio of < 1 compared to Whites for the transitions in our model. We, therefore, treat ethnicity as a binary variable, where ‘0’ represents the reference White group, and ‘1’ represents all other non-White groups. For deprivation, the raw IMD ranges from 1 (least deprived) to 10 (most deprived). In our model, we treated it as a continuous variable, given its ordered nature and for modelling simplicity. For further details on the data extraction, disease and variable definitions, and codelists, we refer to Chen et al.^16^

**Figure 4.**
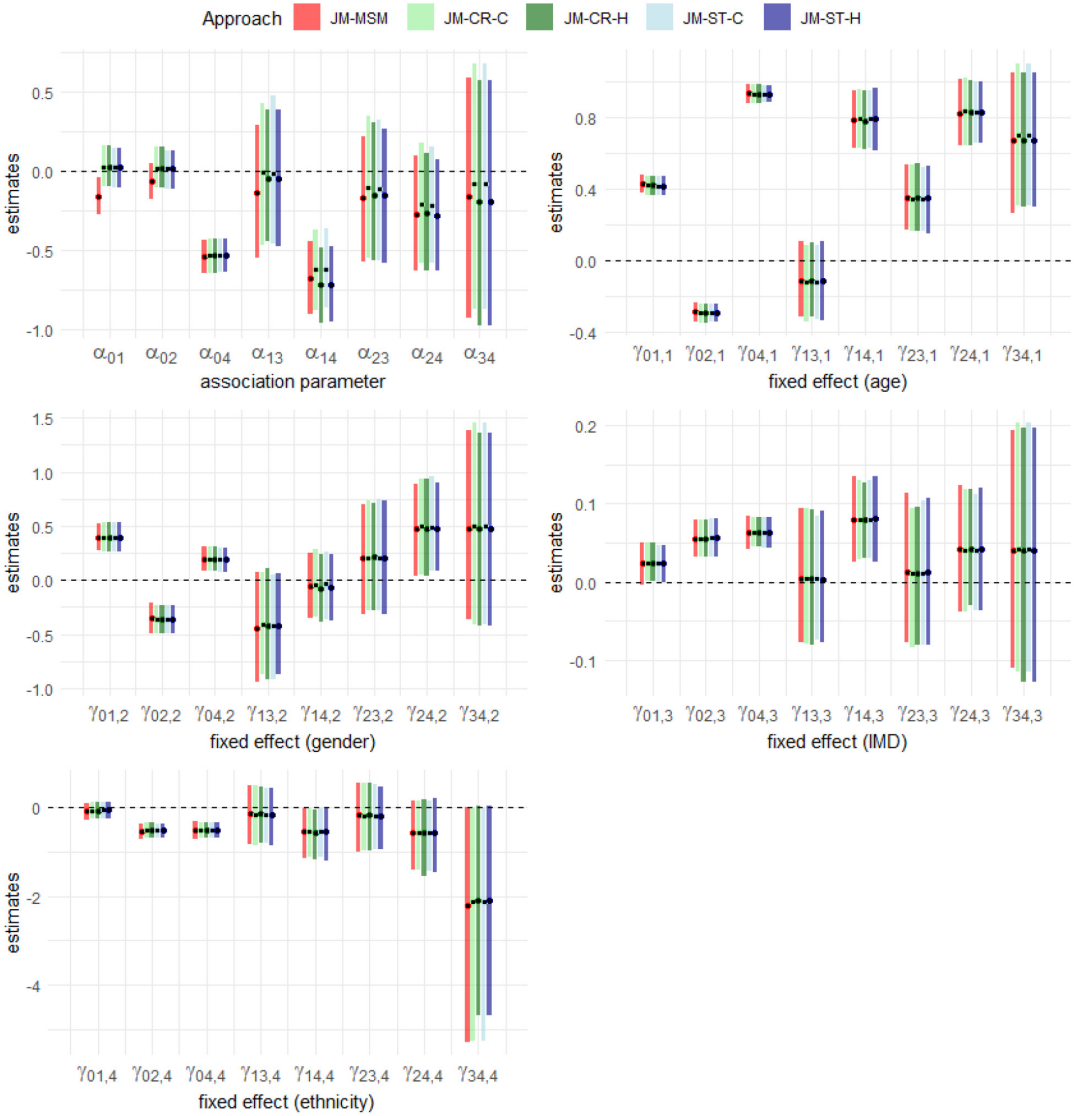
Estimation results for the association parameter ( $\alpha_{jk}$ ) and fixed effects for age (in decades) at entry into the current state ( $\gamma_{jk,1}$ ), gender ( $\gamma_{jk,2}$ ), IMD ( $\gamma_{jk,3}$ ) and ethnicity ( $\gamma_{jk,4}$ ). The results were obtained using the JM-MSM, JM-CR-C, JM-CR-H, JM-ST-C and JM-ST-H approaches, applied to a subset of the CPRD Aurum data with a sample size of n=12,632 . The black dot represents the posterior mean and the superimposed rectangular bar represents the corresponding 95% credible interval. For each parameter, the results from left to right correspond to the JM-MSM, JM-CR-C, JM-CR-H, JM-ST-C and JM-ST-H approaches, respectively. IMD: Index of Multiple Deprivation; CPRD: Clinical Practice Research Datalink; JM-MSM: joint longitudinal and multistate model; JM-CR-C: joint longitudinal and competing risk model (concurrent longitudinal data); JM-ST-C: joint longitudinal and survival model (concurrent longitudinal data); JM-CR-H: joint longitudinal and competing risk model (all historical longitudinal data); JM-ST-H: joint longitudinal and survival model (all historical longitudinal data).

First, we compared the estimation results from the five approaches – JM-MSM, JM-CR-H, JM-CR-C, JM-ST-H and JM-ST-C, as considered in the simulation study. When implementing the blockwise approaches (JM-CR-C and JM-ST-C), historical SBP data were used for inference if a subject lacked an SBP reading associated with the block (see Section 3.3). For this comparison, we used a randomly selected subset of the cohort, consisting of n=12,632 subjects after processing. This sample size is about the largest manageable size for JM-MSM under the computational constraints of the HPC. For all approaches, we used the following set of weakly informative priors: $\alpha_{jk},\gamma_{jk,i},\beta_{i},\eta_{jk,i}∼N(0,10^{2})$ ; $\sigma_{e}^{2},\sigma_{1}^{2}\sigma_{2}^{2}∼\text{Inv-Gamma}(0.01,0.01)$ ; and (ρ+1)/2∼Be(0.5,0.5) ; where $\sigma_{1}^{2}$ and $\sigma_{2}^{2}$ represent the variances of the random effects, ρ is the correlation between the two random effects, and Be(α,β) denotes a Beta distribution with shape parameters α and β . Posterior inference was based on 1000 NUTS samples after a burn-in period of $T_{b}=700$ for JM-MSM and $T_{b}=500$ for JM-CR and JM-ST approaches, selected based on pilot runs and convergence diagnostics. Using the previously specified computing resources, the entire sampling process took approximately 32.1 , 26.2 and 9.3 hours for JM-MSM, JM-CR-C/JM-CR-H and JM-ST-C/JM-ST-H, respectively. As expected, JM-MSM was the most computationally demanding approach, followed by JM-CR, with JM-ST being the most computationally efficient. For this dataset, JM-CR provided a limited computational efficiency gain over JM-MSM, as a significant proportion of subjects were censored within block 1, and this estimation dominated the computing time. Figure 4 displays the posterior means and the associated 95% credible intervals for both the association and fixed effect parameters in the multistate submodel, obtained by each of the five approaches. For the fixed effect parameters, the results are closely aligned across the approaches and are consistent with the findings reported by Chen et al.^16^ For the association parameter, we observe minor discrepancies among the approaches in some transitions. This could be attributed to differences in the amount of longitudinal data used for inference (see Section 3.3). Using the JM-ST approach, we then compared the two data inclusion strategies, JM-ST-C and JM-ST-H, for each transition using the LOO-CV as described in Section 3.4. We consistently found a preference for JM-ST-C over JM-ST-H across all relevant transitions: 1→3 , 1→4 , 2→3 , 2→4 and 3→4 . This result aligns with our impression that JM-ST-C offers a more flexible modelling of the longitudinal trajectory, which can be particularly relevant in complex multistate settings.

Motivated by the comparison results above, we continued the analysis using the JM-ST-C approach to examine the association patterns between SBP and disease transitions with more patients’ data, noting that in Figure 4, estimation results for later stage transitions exhibit high uncertainty due to a lack of data. With the JM-ST approach, we were able to utilise all available data in our cohort for estimating transitions 1→3 , 1→4 , 2→3 , 2→4 and 3→4 , with a total of n=66,490 subjects’ data being used for model fitting (see Supplemental Table 6 for the number of patients’ data used in estimating each transition). For each transition, we further explored three different candidate association structures. Model 1 (M1) includes only the current underlying value of the longitudinal marker SBP. Model 2 (M2) includes the current value of SBP and an interaction effect between age and SBP. Model 3 (M3) extends M1 by adding a quadratic association term, thus allowing for a non-linear relationship between the current value of SBP and the rate of transition. The transition intensities under M1, M2 and M3 are, therefore, given by

$$\begin{array}{rl} \text{M1: }h_{jk}^{\left(i\right)}(t|H_{t^{-}}) & =h_{0,jk}(B(t))\exp\;(w_{i}^{T}\gamma_{jk}+\alpha_{jk}\mu_{i}(t))\\ \text{M2: }h_{jk}^{\left(i\right)}(t|H_{t^{-}}) & =h_{0,jk}(B(t))\exp\;(w_{i}^{T}\gamma_{jk}+\alpha_{jk,1}\mu_{i}(t)+\alpha_{jk,2}({\text{age}}_{j}^{\left(i\right)}\times \mu_{i}(t)))\\ \text{M3: }h_{jk}^{\left(i\right)}(t|H_{t^{-}}) & =h_{0,jk}(B(t))\exp\;(w_{i}^{T}\gamma_{jk}+\alpha_{jk,1}\mu_{i}(t)+\alpha_{jk,2}\mu_{i}^{2}(t)) \end{array}$$

where ${\text{age}}_{j}^{\left(i\right)}$ denotes the age (in decades) of subject i when entering the current state j . Under each model, we implemented the JM-CR-C approach where inference was based on the 1000 NUTS samples following a burn-in of 500 samples (convergence diagnostics based the on trace plots and scale reduction statistic $\hat{R}$ suggest that this is sufficient). We compared M1, M2 and M3 for each transition using the LOO-CV as described in Section 3.4. Supplemental Table 7 shows the posterior summaries of the association parameter(s) obtained under each model, with the favoured model for each transition highlighted in blue shade. It is noteworthy that different association structures were suggested for different types of disease transitions, and for many transitions, the association is significant (as indicated by the fact that the associated 95% credible interval(s) for the association parameter(s) exclude zero). In particular, we observed that a quadratic association pattern is suggested for several transitions, including most of those leading to death. The coefficient of the quadratic effect, $\alpha_{ij,2}$ , maintains a positive value across these transitions, indicating that both lower or higher SBP values are linked with an increased rate of disease progression or mortality. The precise quadratic relationship (controlled by both $\alpha_{ij,1}$ and $\alpha_{ij,2}$ ) varies depending on the subject’s comorbidity status prior to the transition. While it is well established that high SBP is a risk factor for mortality, our finding of an elevated risk at lower SBP levels for multimorbidity progression and mortality is less commonly recognized, and echoes well with recent findings based on Cox regression analysis.^26,27^ Furthermore, the association pattern of SBP level on the rate of transitioning into CVD ( 0→1 and 2→3 ) or MH ( 0→2 and 1→3 ) is found to be influenced by the underlying comorbidity status. For instance, for transitions into CVD, a quadratic association with SBP is identified when T2D is the only existing condition. However, when MH is present as an additional existing comorbidity, an interaction effect between SBP and age is suggested. Our results highlight the complexity of the relationship between a health marker like SBP and the temporal trajectory of multimorbidity.

## 6. Discussion

In this article, we introduce novel yet intuitive blockwise approaches for Bayesian inference in joint longitudinal and MSMs, where the blocks are uniquely determined based on either a competing risks process (JM-CR) or a single transition (JM-ST). Compared to the standard inference approach (JM-MSM), which is based on the posterior distribution of all model parameters conditioned on the full dataset, our proposed blockwise approaches offer enhanced computational efficiency without compromising estimation accuracy. JM-CR enables the inference of both multistate and subject-specific longitudinal parameters. When the interest of inference lies solely in the state transition rates and their associations with the longitudinal process, JM-ST stands out as being the most computationally efficient. For both blockwise approaches, we compared two different strategies for incorporating longitudinal data when performing inference in a block. We found that using only concurrent longitudinal data within the block, when applicable, can be a reasonable choice as it offers more robust inference in the presence of longitudinal model misspecification compared to its counterpart. The practical feasibility and scalability of the blockwise approaches are demonstrated through an application to the CPRD data, where the interest lies in analysing concurrent evolution of SBP and the progression of comorbidities arising from T2D, CVD and MH. Using the JM-ST approach, we were able to utilize data from more patients for inference and efficiently explore different model configurations. Our analysis revealed distinct and previously lesser-known association structures between SBP levels and different disease accumulation trajectories.

The proposed approaches can accommodate broader modelling scenarios than those demonstrated in this article. For instance, multivariate longitudinal processes can be handled using a multivariate GLMM framework,^28^ and nonlinear mixed-effect models can be employed to model the trajectory of specific markers.^29^ More general unidirectional non-Markov multistate processes can also be handled using the same decomposition strategy as considered in Section 3. We anticipate that the computational advantages of using these blockwise approaches will be even more prominent in more complex modelling scenarios. The recent two-stage approach proposed by Alvares and Leiva-Yamaguchi^30^ for JM estimation can also be employed alongside our blockwise approaches to further accelerate the inference in each block. Furthermore, although this article primarily focuses on inference in the multistate process, both JM-CR and JM-ST can be employed to obtain dynamically updated, subject-specific predictions of the risk of transitioning to the next state. This can be achieved either by following the framework described by Ferrer et al.^31^ (if using JM-CR) or by employing a simulation-based approach as described by Crowther and Lambert^24^ (if using JM-ST).

There are several avenues that require future research. In scenarios, where reversible transitions are allowed (i.e. a state can be visited more than once), a subject may have multiple episodes of longitudinal and time-to-event data associated with a specific block. In such cases, additional assumptions need to be introduced to make the blockwise approaches applicable, and their performance in such settings needs to be investigated. In addition, we have focused on scenarios where the multistate process is continuously observed, that is, the exact time of transition is known. However, in some application contexts, these transition times may be subject to interval censoring. This would bring additional computational challenges due to the intractability of the likelihood function for the resulting multistate data.^32^ It would be of interest to explore efficient estimation strategies in these settings.

## Supplemental Material

sj-pdf-1-smm-10.1177_09622802241281959 - Supplemental material for Bayesian blockwise inference for joint models of longitudinal and multistate data with application to longitudinal multimorbidity analysis Supplemental material, sj-pdf-1-smm-10.1177_09622802241281959 for Bayesian blockwise inference for joint models of longitudinal and multistate data with application to longitudinal multimorbidity analysis by Sida Chen, Danilo Alvares, Christopher Jackson, Tom Marshall, Krish Nirantharakumar, Sylvia Richardson, Catherine L Saunders and Jessica K Barrett in Statistical Methods in Medical Research
