# Supplementary material for: Bayesian blockwise inference for joint models of longitudinal and multistate data with application to longitudinal multimorbidity analysis
Source: Stat Methods Med Res. 2024 Oct 21;33(11-12):2027–42. doi: 10.1177/09622802241281959 (PMC11577689; doi:10.1177/09622802241281959)
Supplement: sj-pdf-1-smm-10.1177_09622802241281959 - Supplemental material for Bayesian blockwise inference for joint models of longitudinal and multistate data with application to longitudinal multimorbidity analysis [file sj-pdf-1-smm-10.1177_09622802241281959.pdf]

Supplementary material for "Bayesian blockwise inference for joint models of longitudinal and multistate data with application to longitudinal multimorbidity analysis"

Sida Chen[1], **Danilo Alvares**[1], **Christopher Jackson**[1], **Tom Marshall**[2], **Krish Nirantharakumar**[2], **Sylvia Richardson**[1], **Catherine L. Saunders**[3], **Jessica K. Barrett**[1]

[1] MRC Biostatistics Unit, University of Cambridge, U.K

[2] Institute of Applied Health Research, University of Birmingham, U.K

[3] Department of Public Health and Primary Care, University of Cambridge, U.K

July 23, 2024

A Asymptotic and finite sample comparisons of the inference approaches

Here, we provide a more detailed comparison between the JM-MSM, JM-CR, and JM-ST approaches as described in Sections 2 and 3 of the main paper by examining the full conditional distributions associated with the working posteriors for each approach. These full conditionals are useful for analyzing the corresponding joint posterior density, as the latter is fully characterized by the former according to the Hammersley-Clifford theorem [1]. For ease of notation and comparison, we shall always use all longitudinal data associated with a subject when using JM-CR or JM-ST for blockwise inference. Further, we assume a semi-Markov transition dynamics as considered in the real data application, though the discussion below can carry over to the Markov or other more general scenarios.

Continuing with the notation in Sections 2 and 3 of the main paper, let $\Omega = \{(i,j) \mid i \neq j \in S;\ i \to j \text{ is permitted}\}$. For each type of transition $j \to k$, with $(j,k) \in \Omega$, we introduce a new variable for each subject i as follows. If subject i does not enter state j during the follow-up, we set $D_{jk}^{(i)} = 0$. Otherwise, there will exist an m such that $E_i(T_m^{(i)}) = j$, and we define $D_{jk}^{(i)} = T_{m+1}^{(i)} - T_m^{(i)}$. Let $\delta_{jk}^{(i)}$ be the indicator function associated with $D_{jk}^{(i)}$, where $\delta_{jk}^{(i)} = 1$ if transition $j \to k$ is observed for subject i and is zero if censored (with respect to transition $j \to k$). For a generic competing risk block B_v, and a specific transition within the block $j_v \to k$, where j_v is the initial state in the block and $k \in S_{B_v}$, the working posterior associated with the JM-ST approach is proportional to (see Section 3.2 of the main paper)

$$\prod_{i \in I_{B_v}} f(D_{j_v k}^{(i)}, \delta_{j_v k}^{(i)} \mid \theta_{j_v k}^E, b_i, \theta_y) \prod_{i \in I_{B_v}} \prod_{j=1}^{n_i} f(y_{ij} \mid b_i, \theta_y) f(b_i \mid \theta_b) f(\Theta_{B_{j_v,k}}), \tag{1}$$

where

$$f(D_{jk}^{(i)}, \delta_{jk}^{(i)} \mid \theta_{jk}^E, b_i, \theta_y) = h_{jk}^{(i)}(D_{jk}^{(i)} \mid \theta_{jk}^E, b_i, \theta_y)^{\delta_{jk}^{(i)}} \exp(-\int_0^{D_{jk}^{(i)}} h_{jk}^{(i)}(u \mid \theta_{jk}^E, b_i, \theta_y) du),$$

θ_{jk}^E represents transition-specific MSM parameters, θ_y is the parameter vector associated with the longitudinal submodel, θ_b represents parameters associated with the prior distribution of the random effect, and $\Theta_{B_{j_v,k}} = (\theta_{jk}^E, \theta_y, \theta_b)$. The full conditional distribution for each block of parameters, in the

log-scale, is given by

$$\begin{aligned}
\log f(\theta^E_{j_v k} \mid \cdot) &= \sum_{i \in I_{B_v}} \log f(D^{(i)}_{j_v k}, \delta^{(i)}_{j_v k} \mid \theta^E_{j_v k}, b_i, \theta_y) + \log f(\theta^E_{j_v k}) + C, \\
\log f(b_i \mid \cdot) &= \log f(D^{(i)}_{j_v k}, \delta^{(i)}_{j_v k} \mid \theta^E_{j_v k}, b_i, \theta_y) + \sum_{j=1}^{n_i} \log f(y_{ij} \mid b_i, \theta_y) + \log f(b_i \mid \theta_b) + C, \quad i \in I_{B_v}, \\
\log f(\theta_y \mid \cdot) &= \sum_{i \in I_{B_v}} \log f(D^{(i)}_{j_v k}, \delta^{(i)}_{j_v k} \mid \theta^E_{j_v k}, b_i, \theta_y) + \sum_{i \in I_{B_v}} \sum_{j=1}^{n_i} \log f(y_{ij} \mid b_i, \theta_y) + \log f(\theta_y) + C, \\
\log f(\theta_b \mid \cdot) &= \sum_{i \in I_{B_v}} \log f(b_i \mid \theta_b) + \log f(\theta_b) + C,
\end{aligned} \tag{2}$$

where C denotes a generic constant term (relative to the parameters of interest). The working posterior associated with the JM-CR approach for the block B_v is proportional to (see Section 3.1 of the main paper)

$$\prod_{(j_v,k):k \in S_{B_v}} \prod_{i \in I_{B_v}} f(D^{(i)}_{j_v k}, \delta^{(i)}_{j_v k} \mid \theta^E_{j_v k}, b_i, \theta_y) \prod_{i \in I_{B_v}} \prod_{j=1}^{n_i} f(y_{ij} \mid b_i, \theta_y) f(b_i \mid \theta_b) f(\Theta_{B_v}), \tag{3}$$

where $\Theta_{B_v} = (\{\theta^E_{jk}\}_{k \in S_{B_v}}, \theta_y, \theta_b)$. The associated full conditional distributions are given by

$$\begin{aligned}
&\log f(\theta^E_{j_v k} \mid \cdot) = \sum_{i \in I_{B_v}} \log f(D^{(i)}_{j_v k}, \delta^{(i)}_{j_v k} \mid \theta^E_{j_v k}, b_i, \theta_y) + \log f(\theta^E_{j_v k}) + C, \quad k \in S_{B_v}, \\
&\log f(b_i \mid \cdot) = \sum_{(j_v,k):k \in S_{B_v}} \log f(D^{(i)}_{j_v k}, \delta^{(i)}_{j_v k} \mid \theta^E_{j_v k}, b_i, \theta_y) + \sum_{j=1}^{n_i} \log f(y_{ij} \mid b_i, \theta_y) \\
&+ \log f(b_i \mid \theta_b) + C, \quad i \in I_{B_v}, \\
&\log f(\theta_y \mid \cdot) = \sum_{i \in I_{B_v}} \sum_{(j_v,k):k \in S_{B_v}} \log f(D^{(i)}_{j_v k}, \delta^{(i)}_{j_v k} \mid \theta^E_{j_v k}, b_i, \theta_y) + \sum_{i \in I_{B_v}} \sum_{j=1}^{n_i} \log f(y_{ij} \mid b_i, \theta_y) \\
&+ \log f(\theta_y) + C, \\
&\log f(\theta_b \mid \cdot) = \sum_{i \in I_{B_v}} \log f(b_i \mid \theta_b) + \log f(\theta_b) + C.
\end{aligned} \tag{4}$$

Using the newly introduced variables, the working posterior associated with the JM-MSM approach (see Equations 4 and 5 of the main paper), up to a normalizing constant, can be equivalently expressed as

$$\prod_{(j,k) \in \Omega} \prod_{i=1}^{n} f(D^{(i)}_{jk}, \delta^{(i)}_{jk} \mid \theta^E_{jk}, b_i, \theta_y) \prod_{i=1}^{n} \prod_{j=1}^{n_i} f(y_{ij} \mid b_i, \theta_y) f(b_i \mid \theta_b) f(\Theta), \tag{5}$$

where $\Theta = (\{\theta^E_{jk}\}_{(j,k) \in \Omega}, \theta_y, \theta_b)$. The associated full conditional distributions are given by

$$\begin{aligned}
\log f(\theta^E_{j_v k} \mid \cdot) &= \sum_{i \in I_{B_v}} \log f(D^{(i)}_{j_v k}, \delta^{(i)}_{j_v k} \mid \theta^E_{j_v k}, b_i, \theta_y) + \log f(\theta^E_{j_v k}) + C, \\
\log f(b_i \mid \cdot) &= \sum_{(j,k) \in \Omega} \log f(D^{(i)}_{jk}, \delta^{(i)}_{jk} \mid \theta^E_{jk}, b_i, \theta_y) + \sum_{j=1}^{n_i} \log f(y_{ij} \mid b_i, \theta_y) + \log f(b_i \mid \theta_b) + C, \\
\log f(\theta_y \mid \cdot) &= \sum_{i=1}^{n} \sum_{(j,k) \in \Omega} \log f(D^{(i)}_{jk}, \delta^{(i)}_{jk} \mid \theta^E_{jk}, b_i, \theta_y) + \sum_{i=1}^{n} \sum_{j=1}^{n_i} \log f(y_{ij} \mid b_i, \theta_y) + \log f(\theta_y) + C, \\
\log f(\theta_b \mid \cdot) &= \sum_{i=1}^{n} \log f(b_i \mid \theta_b) + \log f(\theta_b) + C.
\end{aligned} \tag{6}$$

Full conditionals for MSM parameters associated with other types of transitions exhibit a similar form and are therefore omitted here.

We now analyze the behaviours of the marginal posteriors for the transition-specific MSM parameters, θ^E_{jk}, associated with the posteriors as defined in Equations (1), (3), and (5), as n and n_i grow. We proceed by examining the behaviours of the full conditionals associated with (1), (3), and (5). First, note that the full conditional distribution for θ^E_{jk} is exactly the same across three approaches (see the first line of Equations (2), (4), and (6)). With MSM structure fixed (and thus the cardinality of Ω), we observe that as n_i increases, inference from JM-ST and JM-CR would increasingly resemble each other (compare Equations (2) and (4)), as the leading term in $\log f(b_i \mid \cdot)$ and $\log f(\theta_y \mid \cdot)$ is the log-density of the longitudinal submodel, and the full conditional for θ_b is exactly the same under the two approaches. For JM-MSM in (6), the contribution from the longitudinal submodel would also dominate as n_i increases. If in addition, we let n increase, so that the cardinality of I_{B_v} also increases, then under some regularity conditions for the densities of the longitudinal submodel and random effects (which hold for densities in the exponential family), the full conditionals associated with the three approaches would have the same asymptotic distribution according to Bayesian asymptotic theory (see e.g. Chapter 4 of [2] and Chapter 10 of [3]). Therefore, the marginal posterior inference for $\theta^E_{j_v k}$ (and indeed also for θ_y and θ_b) would be asymptotically equivalent under the JM-MSM, JM-CR, and JM-ST approaches. For a given dataset (i.e. fixed n and n_i), the blockwise approaches to posterior inference for a specific block/transition essentially perform inference based solely on the 'relevant' subset of the entire dataset, specifically the data from subjects who are at risk of a transition in the block. As a result, the posterior variability of parameters obtained using blockwise approaches would be expected to be larger than using the JM-MSM approach, which utilizes all data. However, we show in the simulation study that, with a moderate dataset, the blockwise approaches provide good point and interval estimation properties as compared to the JM-MSM approach.

B Additional simulation studies

To assess the performance of the blockwise approaches in comparison to the standard estimation approach for a joint longitudinal and multistate model in the presence of moderate model misspecification, we modify Model 1 of Section 4 in the main paper to create two hypothetical scenarios. In Scenario 1, we modify the underlying subject-specific longitudinal trajectory of Model 1 to incorporate an additional covariate effect, and create a structural change in the trajectory, which occurs immediately after the first transition (into a non-terminal state), i.e., we set

$$\mu_i(t) = \begin{cases} \beta_1 + b_{i1} + \beta_{1,x} w_i + (\beta_2 + b_{i2} + \beta_{2,x} w_i)t & 0 \le t < T_1^{(i)} \\ \beta_1 + b_{i1} + \beta_{1,x} w_i + (\beta_2 + b_{i2} + \beta_{2,x} w_i)t + (\beta_3 + b_{i3})(t - T_1^{(i)}) & t \ge T_1^{(i)} \end{cases} \tag{7}$$

where w_i is the baseline covariate considered in the multistate process of Model 1 and $T_1^{(i)}$ denotes the time of the first transition for individual i. Random effects b_{i1} and b_{i2} are defined as in Model 1 and we set $\beta_1 = 0$, $\beta_2 = 0.5$, $\beta_{1,x} = 0.799$, $\beta_{2,x} = 0.905$ and $\beta_3 = -0.8$. b_{i3} is generated from a Normal distribution with a mean 0 and standard deviation of 0.1, independent of b_{i1} and b_{i2}. This setting is motivated by the likely happening situation where changes may be attributed to changes in the underlying status of the subject or external intervention. For this scenario, the same specification of the multistate process as in Model 1 is used. In Scenario 2, we consider the situation when the multistate process has non-Markov dynamics, where the transition intensities have additional dependency on the dwelling time in the previous state:

$$h^{(i)}_{jk}(t \mid \mathcal{H}_{t^-}) = h_{0,jk}(B(t)) \exp(w_i \gamma_{jk} + \alpha_{jk} \mu_i(t) + \eta_{jk} D^{(i)}_{jk,\text{prev}}), \tag{8}$$

where $(j,k) \in \{(1,3), (1,4), (2,3), (2,4), (3,4)\}$, $D^{(i)}_{jk,\text{prev}}$ denotes the time spent in the previous state, and η_{jk} quantifies the strength of the dependence on $D^{(i)}_{jk,\text{prev}}$. In our simulation, we set $\eta_{13} = 0.1$, $\eta_{14} = -0.2$, $\eta_{23} = 0.2$, $\eta_{24} = -0.3$, and $\eta_{34} = -0.4$. Transitions from state 0 are specified as in Model 1 noting that for these transitions there is no history to consider. For this scenario, the same longitudinal submodel as specified for Model 1 is used.

For each scenario, we generated $N = 200$ replications of the dataset, each with a sample size of $n = 1000$. The five estimation approaches considered in the main simulation study were implemented using the same model specifications and MCMC settings as in Model 1 (for the $n = 1000$ setting), using the previously specified computing resources. Therefore, for all approaches, the longitudinal

submodel is misspecified in Scenario 1 (with the multistate submodel correctly specified for JM-MSM), while in Scenario 2, the multistate submodel is misspecified (with the longitudinal submodel correctly specified for JM-MSM). Figures 5 and 6 summarize the estimated posterior means of the association and other multistate parameters obtained by each approach across the 200 data replications for Scenarios 1 and 2, respectively. In Scenario 1. the JM-MSM, JM-CR-H, and JM-ST-H approaches exhibit significantly larger estimation biases for both types of parameters compared to the JM-CR-C and JM-ST-C approaches. Intuitively, the latter two approaches use 'local' longitudinal data for each block, permitting a more adaptive modelling of the marker's dynamics and making them more reflective of any structural changes in the trajectory. In addition, the satisfactory estimation accuracy of JM-CR-C and JM-ST-C suggests that the omission of covariate(s) in the longitudinal submodel has minimal influence on the estimation of multistate parameters under the current value association. In Scenario 2, all five approaches produce similar estimation results, with varying levels of estimation biases depending on the transition types and parameter types. The results suggest that in the presence of misspecified transition intensities, using blockwise approaches does not incur higher biases compared to JM-MSM when all are set up with the same model specification.

References

[1] Robert CP, Casella G and Casella G. *Monte Carlo statistical methods*, volume 2. Springer, 1999.

[2] Gelman A, Carlin JB, Stern HS et al. *Bayesian data analysis*. CRC press, 2013.

[3] Cox DR and Hinkley DV. *Theoretical statistics*. CRC Press, 1979.

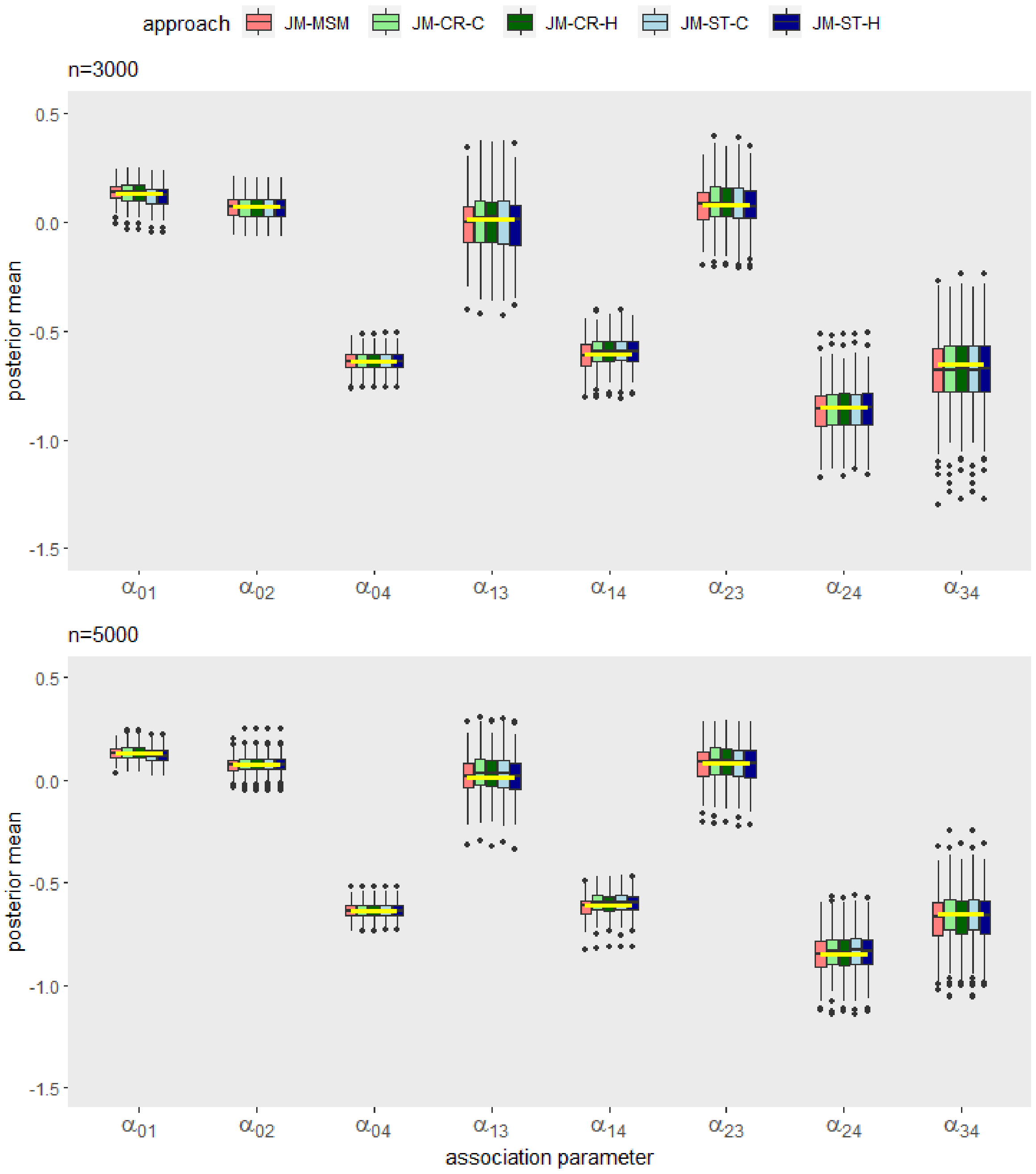

Figure 1: Box plot summary of the posterior mean of the association parameter α_{jk} obtained by each approach from 200 replications of the data simulated from Model 1. Rows 1 and 2 show the results for $n = 3000$ and $n = 5000$, respectively. The true parameter value for each α_{jk} is indicated by a yellow horizontal bar.

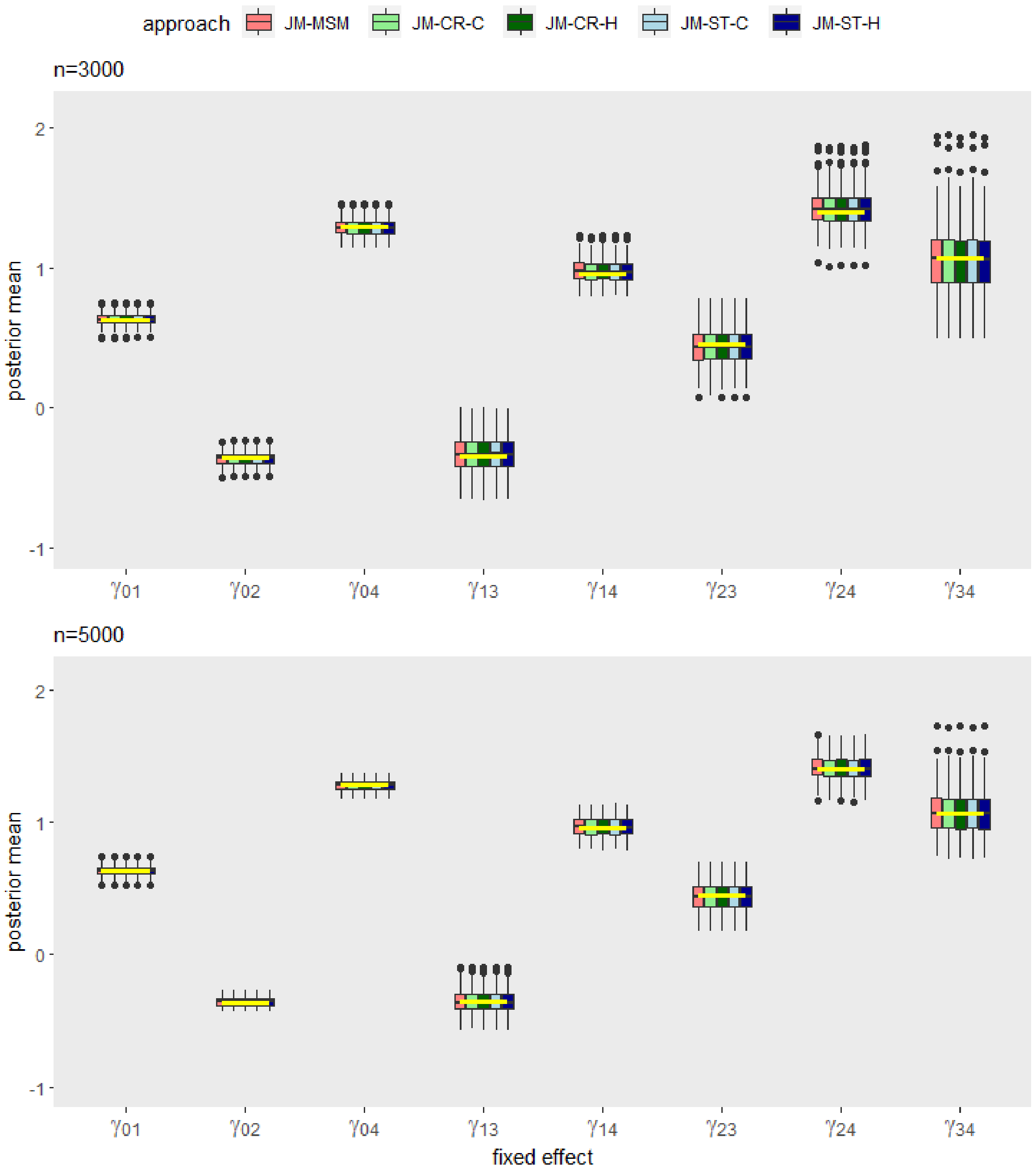

Figure 2: Box plot summary of the posterior mean of the fixed effect γ_{jk} obtained by each approach from 200 replications of the data simulated from Model 1. Rows 1 and 2 show the results for $n = 3000$ and $n = 5000$, respectively. The true parameter value for each γ_{jk} is indicated by a yellow horizontal bar.

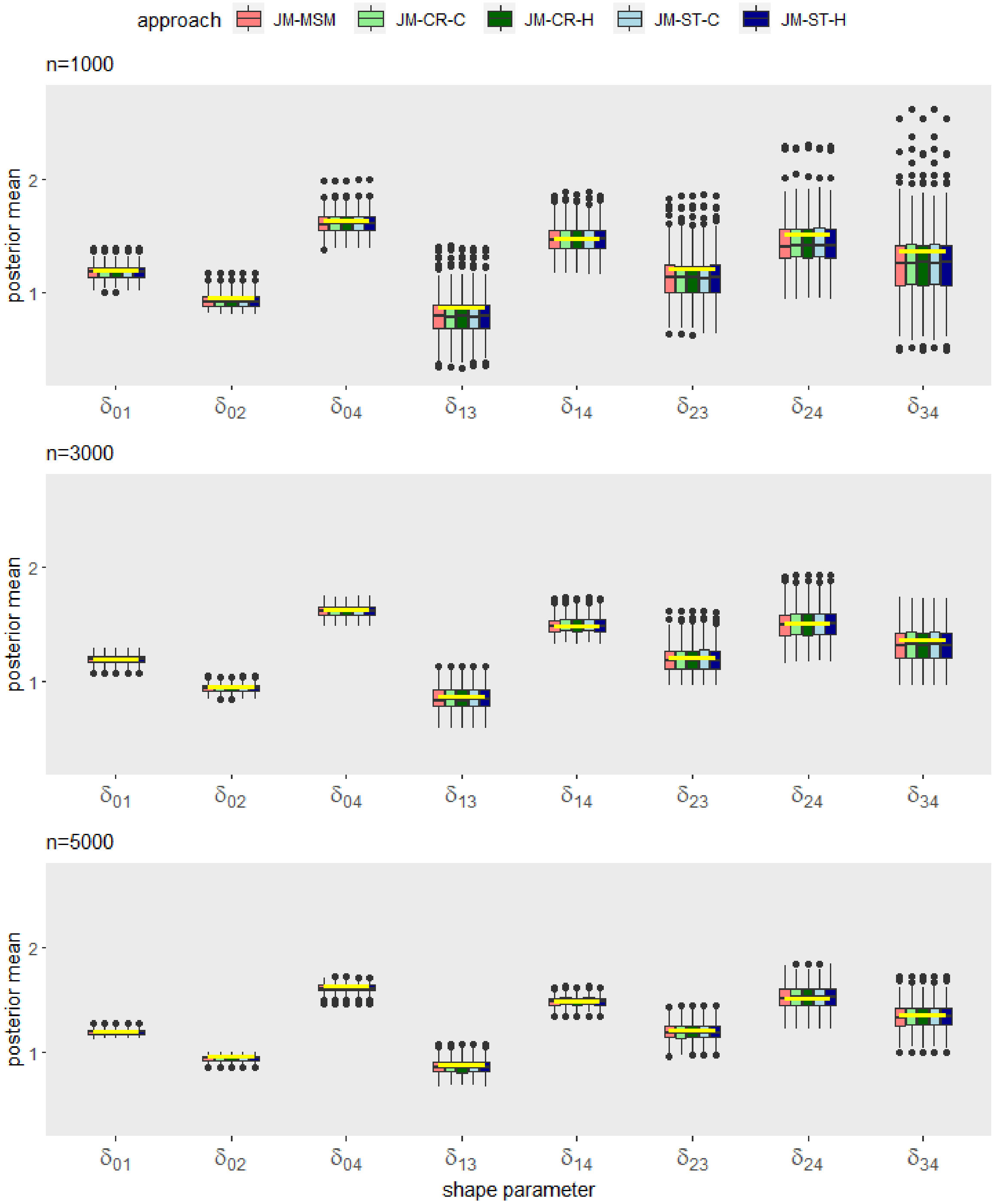

Figure 3: Box plot summary of the posterior mean of the Weibull shape parameter δ_{jk} obtained by each approach from 200 replications of the data simulated from Model 1. Rows 1, 2 and 3 show the results for $n = 1000$, $n = 3000$ and $n = 5000$, respectively. The true parameter value for each δ_{jk} is indicated by a yellow horizontal bar.

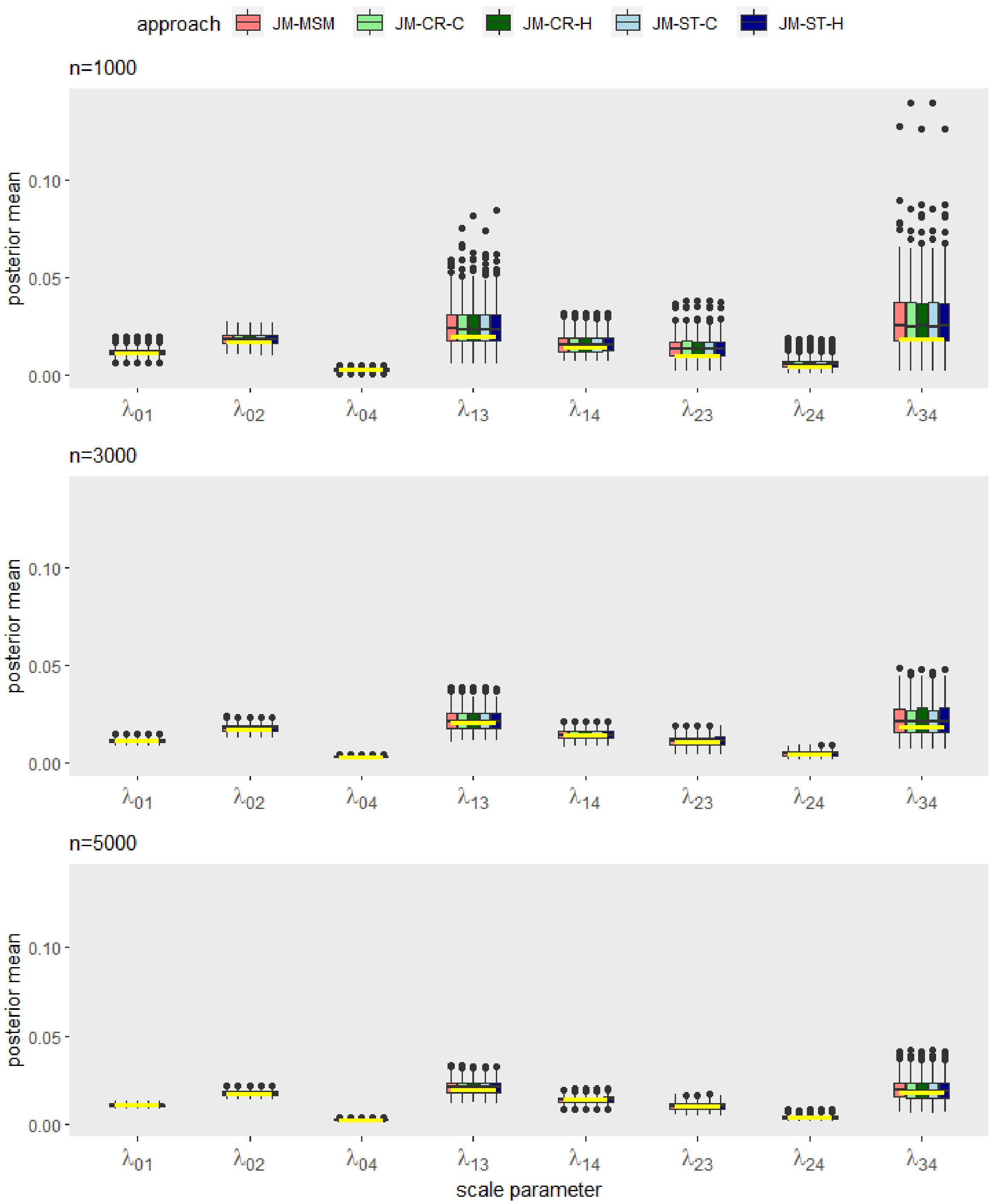

Figure 4: Box plot summary of the posterior mean of the Weibull scale parameter λ_{jk} obtained by each approach from 200 replications of the data simulated from Model 1. Rows 1, 2 and 3 show the results for $n = 1000$, $n = 3000$ and $n = 5000$, respectively. The true parameter value for each λ_{jk} is indicated by a yellow horizontal bar.

Table 1: Coverage probability of estimated 95% credible interval for the association parameter based on 200 replications of data from simulation Model 1.

Parameter	Approach	$n = 1000$	$n = 3000$	$n = 5000$
	JM-MSM	0.950	0.965	0.965
	JM-CR-C	0.955	0.980	0.910
α_{01}	JM-CR-H	0.955	0.980	0.910
	JM-ST-C	0.955	0.945	0.910
	JM-ST-H	0.955	0.945	0.910
	JM-MSM	0.950	0.940	0.935
	JM-CR-C	0.940	0.945	0.915
α_{02}	JM-CR-H	0.940	0.945	0.915
	JM-ST-C	0.945	0.945	0.910
	JM-ST-H	0.945	0.945	0.910
	JM-MSM	0.940	0.960	0.945
	JM-CR-C	0.930	0.955	0.960
α_{04}	JM-CR-H	0.930	0.955	0.960
	JM-ST-C	0.930	0.955	0.960
	JM-ST-H	0.930	0.955	0.960
	JM-MSM	0.970	0.940	0.925
	JM-CR-C	0.970	0.955	0.945
α_{13}	JM-CR-H	0.985	0.950	0.940
	JM-ST-C	0.975	0.945	0.930
	JM-ST-H	0.985	0.930	0.950
	JM-MSM	0.975	0.940	0.945
	JM-CR-C	0.970	0.935	0.955
α_{14}	JM-CR-H	0.970	0.940	0.940
	JM-ST-C	0.965	0.935	0.960
	JM-ST-H	0.970	0.945	0.950
	JM-MSM	0.960	0.965	0.930
	JM-CR-C	0.960	0.940	0.940
α_{23}	JM-CR-H	0.950	0.955	0.950
	JM-ST-C	0.960	0.945	0.950
	JM-ST-H	0.950	0.940	0.950
	JM-MSM	0.950	0.945	0.925
	JM-CR-C	0.965	0.965	0.925
α_{24}	JM-CR-H	0.945	0.955	0.920
	JM-ST-C	0.965	0.960	0.930
	JM-ST-H	0.950	0.955	0.930
	JM-MSM	0.935	0.955	0.925
	JM-CR-C	0.935	0.955	0.930
α_{34}	JM-CR-H	0.940	0.960	0.945
	JM-ST-C	0.935	0.955	0.930
	JM-ST-H	0.940	0.960	0.945

Table 2: Coverage probability of estimated 95% credible interval for the fixed effect based on 200 replications of data from simulation Model 1.

Parameter	Approach	$n = 1000$	$n = 3000$	$n = 5000$
γ_{01}	JM-MSM	0.980	0.945	0.950
	JM-CR-C	0.985	0.945	0.960
	JM-CR-H	0.985	0.945	0.960
	JM-ST-C	0.980	0.945	0.950
	JM-ST-H	0.980	0.945	0.950
γ_{02}	JM-MSM	0.940	0.925	0.960
	JM-CR-C	0.945	0.935	0.950
	JM-CR-H	0.945	0.935	0.950
	JM-ST-C	0.930	0.920	0.970
	JM-ST-H	0.930	0.920	0.970
γ_{04}	JM-MSM	0.950	0.945	0.965
	JM-CR-C	0.940	0.950	0.970
	JM-CR-H	0.940	0.950	0.970
	JM-ST-C	0.950	0.940	0.970
	JM-ST-H	0.950	0.940	0.970
γ_{13}	JM-MSM	0.935	0.940	0.960
	JM-CR-C	0.950	0.940	0.955
	JM-CR-H	0.940	0.955	0.960
	JM-ST-C	0.920	0.935	0.960
	JM-ST-H	0.960	0.935	0.965
γ_{14}	JM-MSM	0.945	0.950	0.930
	JM-CR-C	0.935	0.970	0.940
	JM-CR-H	0.950	0.965	0.935
	JM-ST-C	0.930	0.950	0.950
	JM-ST-H	0.935	0.945	0.925
γ_{23}	JM-MSM	0.960	0.930	0.940
	JM-CR-C	0.955	0.930	0.920
	JM-CR-H	0.950	0.935	0.925
	JM-ST-C	0.960	0.930	0.920
	JM-ST-H	0.950	0.940	0.920
γ_{24}	JM-MSM	0.965	0.960	0.975
	JM-CR-C	0.970	0.955	0.970
	JM-CR-H	0.960	0.955	0.965
	JM-ST-C	0.955	0.950	0.975
	JM-ST-H	0.955	0.955	0.965
γ_{34}	JM-MSM	0.945	0.945	0.955
	JM-CR-C	0.940	0.945	0.960
	JM-CR-H	0.940	0.945	0.965
	JM-ST-C	0.940	0.945	0.960
	JM-ST-H	0.940	0.945	0.965

Table 3: Coverage probability of estimated 95% credible interval for the shape parameter based on 200 replications of data from simulation Model 1.

Parameter	Approach	$n = 1000$	$n = 3000$	$n = 5000$
δ_{01}	JM-MSM	0.975	0.975	0.950
	JM-CR-C	0.970	0.975	0.955
	JM-CR-H	0.970	0.975	0.955
	JM-ST-C	0.975	0.975	0.940
	JM-ST-H	0.975	0.975	0.940
δ_{02}	JM-MSM	0.930	0.955	0.930
	JM-CR-C	0.935	0.960	0.925
	JM-CR-H	0.935	0.960	0.925
	JM-ST-C	0.920	0.965	0.925
	JM-ST-H	0.920	0.965	0.925
δ_{04}	JM-MSM	0.945	0.925	0.890
	JM-CR-C	0.940	0.925	0.880
	JM-CR-H	0.940	0.925	0.880
	JM-ST-C	0.935	0.930	0.885
	JM-ST-H	0.935	0.930	0.885
δ_{13}	JM-MSM	0.915	0.940	0.945
	JM-CR-C	0.910	0.930	0.950
	JM-CR-H	0.910	0.925	0.945
	JM-ST-C	0.910	0.930	0.945
	JM-ST-H	0.910	0.930	0.940
δ_{14}	JM-MSM	0.920	0.940	0.950
	JM-CR-C	0.920	0.945	0.955
	JM-CR-H	0.915	0.950	0.960
	JM-ST-C	0.915	0.940	0.940
	JM-ST-H	0.905	0.925	0.945
δ_{23}	JM-MSM	0.895	0.940	0.950
	JM-CR-C	0.890	0.935	0.955
	JM-CR-H	0.875	0.945	0.950
	JM-ST-C	0.880	0.930	0.955
	JM-ST-H	0.885	0.935	0.950
δ_{24}	JM-MSM	0.930	0.930	0.955
	JM-CR-C	0.930	0.930	0.955
	JM-CR-H	0.935	0.935	0.955
	JM-ST-C	0.930	0.920	0.945
	JM-ST-H	0.935	0.930	0.955
δ_{34}	JM-MSM	0.940	0.935	0.950
	JM-CR-C	0.935	0.940	0.950
	JM-CR-H	0.945	0.940	0.950
	JM-ST-C	0.935	0.940	0.950
	JM-ST-H	0.945	0.940	0.950

Table 4: Coverage probability of estimated 95% credible interval for the scale parameter based on 200 replications of data from simulation Model 1.

Parameter	Approach	$n = 1000$	$n = 3000$	$n = 5000$
	JM-MSM	0.950	0.980	0.955
	JM-CR-C	0.955	0.985	0.970
λ_{01}	JM-CR-H	0.955	0.985	0.970
	JM-ST-C	0.955	0.980	0.960
	JM-ST-H	0.955	0.980	0.960
	JM-MSM	0.950	0.950	0.945
	JM-CR-C	0.945	0.935	0.940
λ_{02}	JM-CR-H	0.945	0.935	0.940
	JM-ST-C	0.935	0.950	0.940
	JM-ST-H	0.935	0.950	0.940
	JM-MSM	0.950	0.930	0.905
	JM-CR-C	0.950	0.920	0.885
λ_{04}	JM-CR-H	0.950	0.920	0.885
	JM-ST-C	0.950	0.935	0.885
	JM-ST-H	0.950	0.935	0.885
	JM-MSM	0.925	0.955	0.940
	JM-CR-C	0.925	0.930	0.940
λ_{13}	JM-CR-H	0.925	0.935	0.945
	JM-ST-C	0.915	0.940	0.940
	JM-ST-H	0.925	0.945	0.935
	JM-MSM	0.930	0.965	0.945
	JM-CR-C	0.935	0.960	0.935
λ_{14}	JM-CR-H	0.940	0.965	0.940
	JM-ST-C	0.925	0.955	0.935
	JM-ST-H	0.935	0.960	0.940
	JM-MSM	0.940	0.950	0.975
	JM-CR-C	0.940	0.950	0.965
λ_{23}	JM-CR-H	0.955	0.945	0.960
	JM-ST-C	0.940	0.945	0.975
	JM-ST-H	0.935	0.940	0.975
	JM-MSM	0.960	0.950	0.970
	JM-CR-C	0.950	0.955	0.970
λ_{24}	JM-CR-H	0.945	0.945	0.965
	JM-ST-C	0.940	0.955	0.960
	JM-ST-H	0.945	0.950	0.965
	JM-MSM	0.950	0.965	0.945
	JM-CR-C	0.955	0.960	0.935
λ_{34}	JM-CR-H	0.950	0.965	0.925
	JM-ST-C	0.955	0.960	0.935
	JM-ST-H	0.950	0.965	0.925

Table 5: Coverage probability of estimated 95% credible interval for the association and fixed effect parameters based on 200 replications of data from simulation Model 2 ($n = 900$).

Parameter	Approach	Coverage probability (α_{jk})	Parameter	Approach	Coverage probability (γ_{jk})
	JM-MSM	0.945		JM-MSM	0.935
	JM-CR-C	0.950		JM-CR-C	0.940
α_{01}	JM-CR-H	0.950	γ_{01}	JM-CR-H	0.940
	JM-ST-C	0.950		JM-ST-C	0.930
	JM-ST-H	0.950		JM-ST-H	0.930
	JM-MSM	0.945		JM-MSM	0.970
	JM-CR-C	0.960		JM-CR-C	0.965
α_{02}	JM-CR-H	0.960	γ_{02}	JM-CR-H	0.965
	JM-ST-C	0.950		JM-ST-C	0.970
	JM-ST-H	0.950		JM-ST-H	0.970
	JM-MSM	0.950		JM-MSM	0.935
	JM-CR-C	0.940		JM-CR-C	0.935
α_{03}	JM-CR-H	0.940	γ_{03}	JM-CR-H	0.935
	JM-ST-C	0.945		JM-ST-C	0.925
	JM-ST-H	0.945		JM-ST-H	0.925
	JM-MSM	0.930		JM-MSM	0.965
	JM-CR-C	0.925		JM-CR-C	0.970
α_{04}	JM-CR-H	0.925	γ_{04}	JM-CR-H	0.970
	JM-ST-C	0.920		JM-ST-C	0.965
	JM-ST-H	0.920		JM-ST-H	0.965
	JM-MSM	0.975		JM-MSM	0.940
	JM-CR-C	0.980		JM-CR-C	0.960
α_{12}	JM-CR-H	0.975	γ_{12}	JM-CR-H	0.945
	JM-ST-C	0.980		JM-ST-C	0.950
	JM-ST-H	0.975		JM-ST-H	0.955
	JM-MSM	0.940		JM-MSM	0.925
	JM-CR-C	0.935		JM-CR-C	0.920
α_{13}	JM-CR-H	0.925	γ_{13}	JM-CR-H	0.900
	JM-ST-C	0.940		JM-ST-C	0.915
	JM-ST-H	0.930		JM-ST-H	0.920
	JM-MSM	0.945		JM-MSM	0.960
	JM-CR-C	0.950		JM-CR-C	0.960
α_{14}	JM-CR-H	0.950	γ_{14}	JM-CR-H	0.965
	JM-ST-C	0.950		JM-ST-C	0.965
	JM-ST-H	0.960		JM-ST-H	0.970
	JM-MSM	0.965		JM-MSM	0.950
	JM-CR-C	0.975		JM-CR-C	0.950
α_{23}	JM-CR-H	0.970	γ_{23}	JM-CR-H	0.960
	JM-ST-C	0.980		JM-ST-C	0.960
	JM-ST-H	0.975		JM-ST-H	0.945
	JM-MSM	0.915		JM-MSM	0.925
	JM-CR-C	0.925		JM-CR-C	0.935
α_{24}	JM-CR-H	0.915	γ_{24}	JM-CR-H	0.930
	JM-ST-C	0.915		JM-ST-C	0.935
	JM-ST-H	0.910		JM-ST-H	0.930
	JM-MSM	0.935		JM-MSM	0.945
	JM-CR-C	0.935		JM-CR-C	0.945
α_{34}	JM-CR-H	0.935	γ_{34}	JM-CR-H	0.950
	JM-ST-C	0.935		JM-ST-C	0.945
	JM-ST-H	0.935		JM-ST-H	0.950

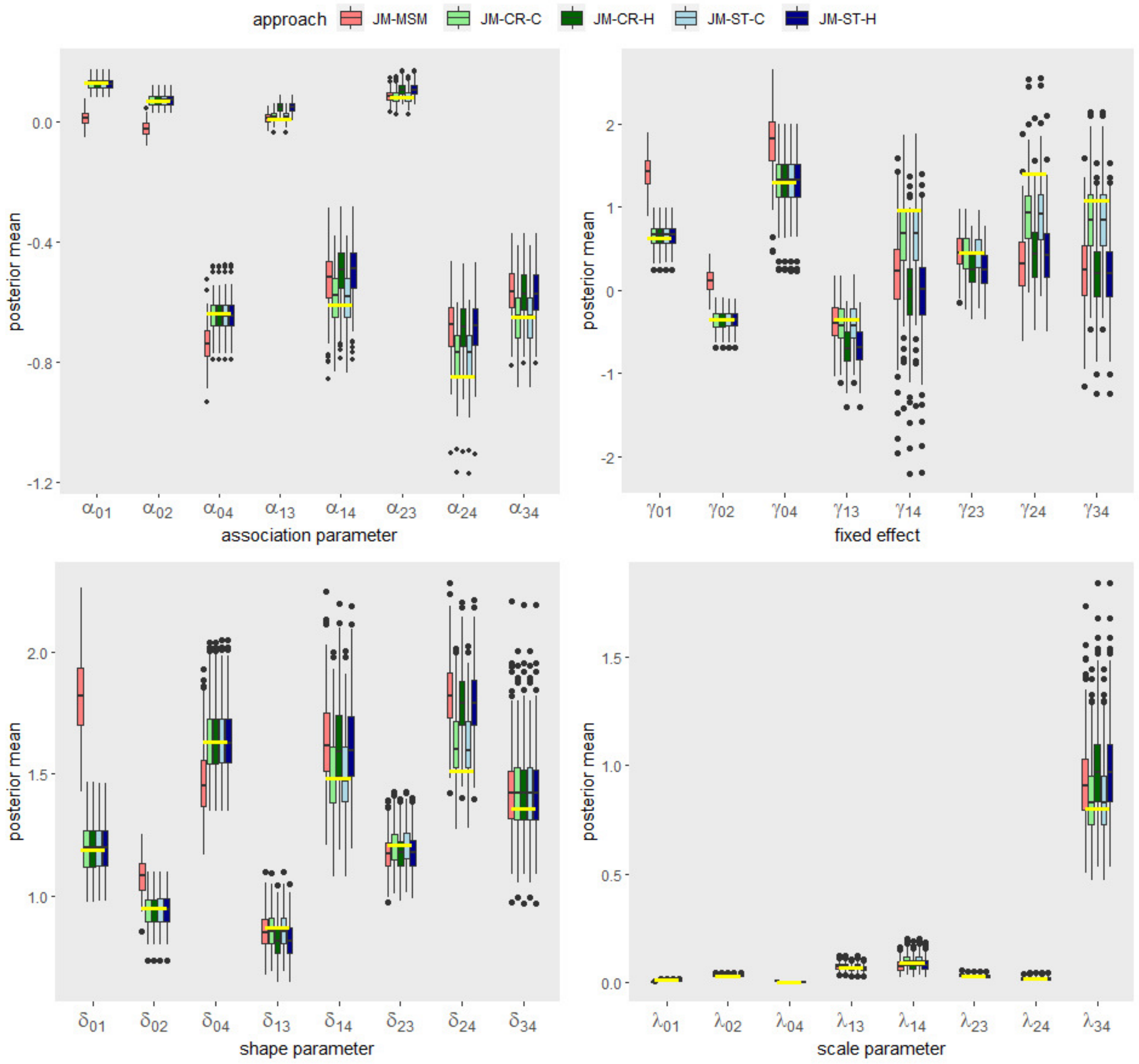

Figure 5: Box plot summary of the posterior means of the association and other multistate parameters obtained by each approach from 200 replications of the data simulated from Scenario 1 in Section B. The true values for each parameter are indicated by yellow horizontal bars.

Table 6: Number of subjects' data used for estimating each disease transition with the JM-ST-C approach in the CPRD application, as described in Section 5 of the main paper.

**Transition**	$0 \to 1$, $0 \to 2$, $0 \to 4$	$1 \to 3$	$1 \to 4$	$2 \to 3$	$2 \to 4$	$3 \to 4$
**Sample size**	30634	20018	20017	19903	19901	2647

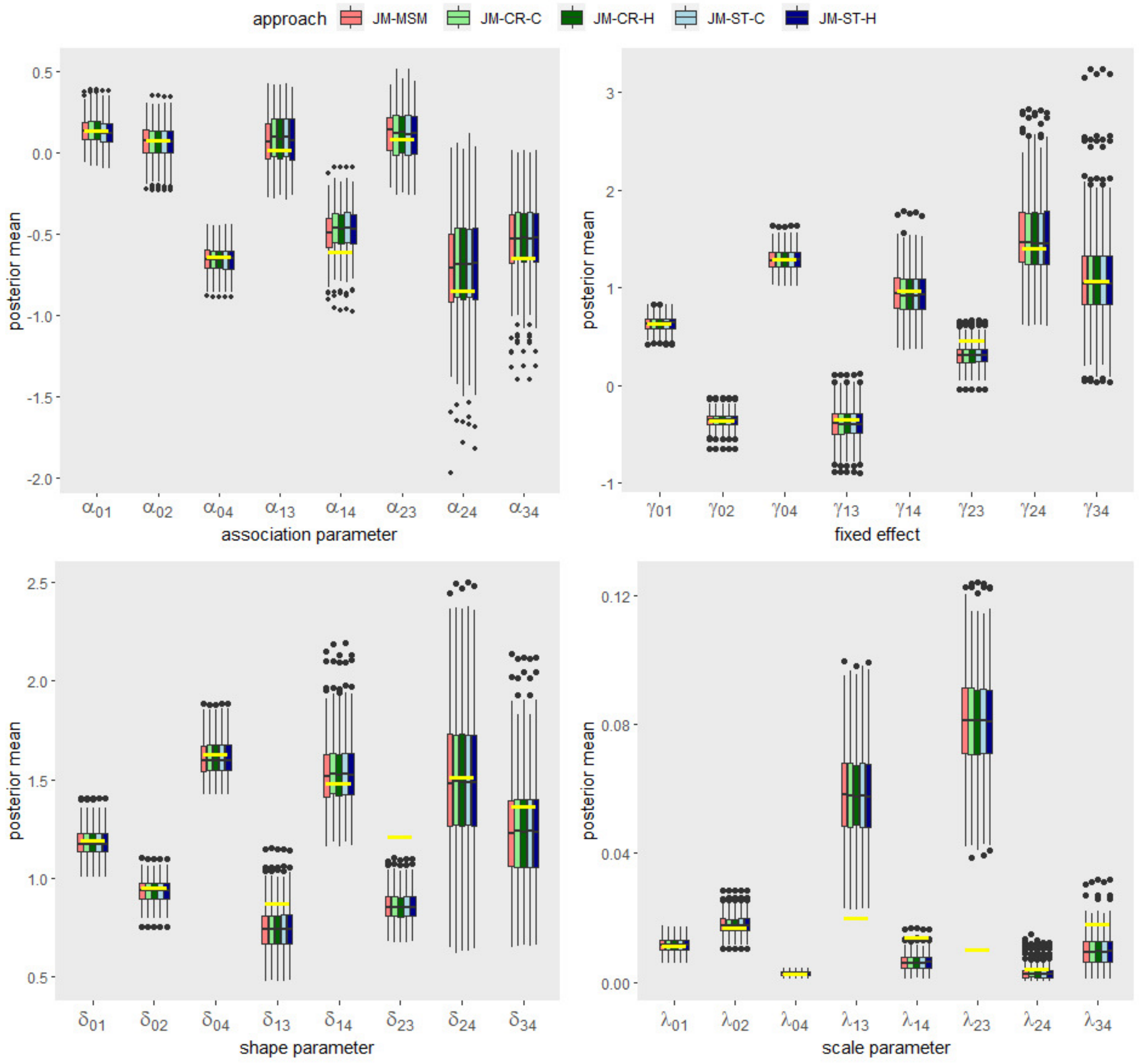

Figure 6: Box plot summary of the posterior means of the association and other multistate parameters obtained by each approach from 200 replications of the data simulated from Scenario 2 in Section B. The true values for each parameter are indicated by yellow horizontal bars.

Table 7: Posterior summary (mean and 95% credible interval) of the association parameter estimated using JM-ST-C approach under models M1, M2, and M3 in the CPRD application, as described in Section 5 of the main paper. The favoured model based on the LOO-CV for each transition is highlighted in blue shade.

M1 (current SBP value)				
Disease Transition	Parameter	Mean	2.5%	97.5%
T2D → T2D+CVD	α_{01}	-0.005	-0.087	0.073
T2D → T2D+MH	α_{02}	-0.060	-0.143	0.030
T2D → Death	α_{04}	-0.553	-0.625	-0.487
T2D+CVD → T2D+CVD+MH	α_{13}	0.083	-0.019	0.180
T2D+CVD → Death	α_{14}	-0.412	-0.460	-0.363
T2D+MH → T2D+MH+CVD	α_{23}	0.130	0.028	0.231
T2D+MH → Death	α_{24}	-0.502	-0.575	-0.425
T2D+MH+CVD → Death	$\boldsymbol{\alpha_{34}}$	**-0.404**	**-0.552**	**-0.248**
M2 (extends M1 with age-SBP interaction)				
Disease Transition	Parameter	Mean	2.5%	97.5%
T2D → T2D+CVD	$\alpha_{01,1}$	0.343	-0.048	0.737
	$\alpha_{01,2}$	-0.053	-0.113	0.004
T2D → T2D+MH	$\alpha_{02,1}$	0.126	-0.196	0.449
	$\alpha_{02,2}$	-0.035	-0.095	0.020
T2D → Death	$\alpha_{04,1}$	-0.514	-0.957	-0.072
	$\alpha_{04,2}$	-0.005	-0.066	0.057
T2D+CVD → T2D+CVD+MH	$\boldsymbol{\alpha_{13,1}}$	**0.132**	**-0.389**	**0.648**
	$\boldsymbol{\alpha_{13,2}}$	**-0.008**	**-0.084**	**0.072**
T2D+CVD → Death	$\alpha_{14,1}$	-0.911	-1.288	-0.491
	$\alpha_{14,2}$	0.064	0.011	0.112
T2D+MH → T2D+MH+CVD	$\boldsymbol{\alpha_{23,1}}$	**0.515**	**0.050**	**0.990**
	$\boldsymbol{\alpha_{23,2}}$	**-0.060**	**-0.135**	**0.015**
T2D+MH → Death	$\alpha_{24,1}$	-0.281	-0.716	0.170
	$\alpha_{24,2}$	-0.030	-0.092	0.028
T2D+MH+CVD → Death	$\alpha_{34,1}$	0.817	-0.153	1.809
	$\alpha_{34,2}$	-0.161	-0.291	-0.032
M3 (extends M1 with quadratic SBP term)				
Disease Transition	Parameter	Mean	2.5%	97.5%
T2D → T2D+CVD	$\boldsymbol{\alpha_{01,1}}$	**0.085**	**0.012**	**0.160**
	$\boldsymbol{\alpha_{01,2}}$	**0.201**	**0.138**	**0.258**
T2D → T2D+MH	$\boldsymbol{\alpha_{02,1}}$	**-0.033**	**-0.123**	**0.055**
	$\boldsymbol{\alpha_{02,2}}$	**0.048**	**-0.036**	**0.123**
T2D → Death	$\boldsymbol{\alpha_{04,1}}$	**-0.335**	**-0.407**	**-0.261**
	$\boldsymbol{\alpha_{04,2}}$	**0.230**	**0.173**	**0.284**
T2D+CVD → T2D+CVD+MH	$\alpha_{13,1}$	0.125	0.000	0.243
	$\alpha_{13,2}$	0.067	-0.038	0.167
T2D+CVD → Death	$\boldsymbol{\alpha_{14,1}}$	**-0.170**	**-0.237**	**-0.105**
	$\boldsymbol{\alpha_{14,2}}$	**0.229**	**0.185**	**0.272**
T2D+MH → T2D+MH+CVD	$\alpha_{23,1}$	0.197	0.111	0.281
	$\alpha_{23,2}$	0.267	0.193	0.342
T2D+MH → Death	$\boldsymbol{\alpha_{24,1}}$	**-0.298**	**-0.377**	**-0.222**
	$\boldsymbol{\alpha_{24,2}}$	**0.279**	**0.219**	**0.339**
T2D+MH+CVD → Death	$\alpha_{34,1}$	-0.200	-0.366	-0.047
	$\alpha_{34,2}$	0.237	0.087	0.371
